# Neural progenitor–derived Apelin controls tip cell behavior and vascular patterning

**DOI:** 10.1126/sciadv.adk1174

**Published:** 2024-07-05

**Authors:** Julian Malchow, Jean Eberlein, Wei Li, Benjamin M. Hogan, Kazuhide S. Okuda, Christian S. M. Helker

**Affiliations:** ^1^ Faculty of Biology, Cell Signaling and Dynamics, Philipps-University of Marburg, Marburg, Germany.; ^2^ Organogenesis and Cancer Program, Peter MacCallum Cancer Centre, Melbourne, Victoria, Australia.; ^3^ Sir Peter MacCallum Department of Oncology, University of Melbourne, Melbourne, Victoria 3000, Australia.; ^4^ Department of Anatomy and Physiology, University of Melbourne, Melbourne, Victoria 3000, Australia.; ^5^ Department of Biochemistry and Chemistry, School of Agriculture, Biomedicine and Environment, La Trobe University, Melbourne, Victoria, Australia.; ^6^ Centre for Cardiovascular Biology and Disease Research, La Trobe Institute for Molecular Science, La Trobe University, Melbourne, Victoria, Australia.; ^7^ Center for Mind, Brain and Behavior (CMBB), University of Marburg and Justus-Liebig-University Giessen, Marburg, Germany.

## Abstract

During angiogenesis, vascular tip cells guide nascent vascular sprouts to form a vascular network. Apelin, an agonist of the G protein–coupled receptor Aplnr, is enriched in vascular tip cells, and it is hypothesized that vascular-derived Apelin regulates sprouting angiogenesis. We identify an *apelin*-expressing neural progenitor cell population in the dorsal neural tube. Vascular tip cells exhibit directed elongation and migration toward and along the *apelin*-expressing neural progenitor cells. Notably, restoration of neural but not vascular *apelin* expression in *apelin* mutants remedies the angiogenic defects of mutants. By functional analyses, we show the requirement of Apelin signaling for tip cell behaviors, like filopodia formation and cell elongation. Through genetic interaction studies and analysis of transgenic activity reporters, we identify Apelin signaling as a modulator of phosphoinositide 3-kinase and extracellular signal–regulated kinase signaling in tip cells in vivo. Our results suggest a previously unidentified neurovascular cross-talk mediated by Apelin signaling that is important for tip cell function during sprouting angiogenesis.

## Teaser

An Apelin signaling–mediated neurovascular cross-talk drives endothelial tip cell function and spinal cord vascularization.

## INTRODUCTION

To establish a three-dimensional vascular network that sustains organ function, endothelial cells (ECs) leave preexisting vessels and migrate into avascular areas in a process termed sprouting angiogenesis. Vascular endothelial growth factor (VEGF) signaling initiates angiogenesis by triggering individual ECs to sprout from neighboring vessels. Subsequently, trailing cells follow leading cells, resulting in the formation of a sprout with specialized tip cells at the forefront and stalk cells trailing behind (*1*). Within the vascular sprouts, ECs dynamically compete for the tip cell position (*2*). Tip cells, characterized by dynamic filopodia extensions, sense secreted molecules such as VEGF-A (*1*, *3*). Zebrafish embryos deficient for *vegfaa* or the Vegfr2 paralog *kdrl* exhibit severe defects of intersegmental vessel (ISV) outgrowth, with delayed budding of tip cells and absence of most ISVs (*4*, *5*). Increased VEGF signaling levels in tip cells lead to elevated extracellular signal–regulated kinase (ERK) activity and DLL4 (Delta-like 4) expression, which inhibits tip cell fate in stalk cells through Notch receptor signaling (*6*–*9*). In addition, asymmetric cell divisions resulting in larger tip daughter cells compared to stalk daughter cells maintain high VEGF signaling in tip cells (*10*).

While recent studies have elucidated the importance of the Apelin signaling pathway in angiogenesis across various species and in different contexts (*11*–*19*), the specific mechanisms governing its regulation of tip cell behavior and migration remain unknown. The Apelin receptor (Aplnr), a G protein–coupled receptor (GPCR), is expressed in angiogenic ECs (*11*), whereas its ligand Apelin (Apln) is enriched in tip cells (*11*, *20*). In zebrafish, the Apelin signaling pathway is composed of two ligands, Apela (also known as Elabela or Toddler) and Apln, along with two receptors Aplnra and Aplnrb. Previously, it has been shown that Apela is expressed in the early embryo and regulates mesodermal cell movements (*21*) and angioblast migration (*15*). Yet, our previous findings show that the development of the ISVs mainly depends on Apln and Aplnrb (*11*). Moreover, zebrafish mutants for *apln* or *aplnrb* exhibit defects in the morphology of tip cells and their migration (*11*). However, the exact molecular and cellular mechanisms by which Apelin signaling regulates endothelial tip cell behavior and migration are not known.

In this study, we use state-of-the-art confocal time-lapse imaging of developing zebrafish embryos, coupled with novel transgenic reporters and genetic experiments, to unravel a previously undiscovered neurovascular cross-talk mediated by Apelin signaling. Our findings reveal that Apelin signals originate from neural progenitor cells in the dorsal neural tube and are transmitted to the tip cell. Neural progenitor–derived Apelin guides tip cell migration toward the neural tube, facilitating the formation of the dorsal longitudinal anastomotic vessel (DLAV) by directing migration of tip cells along *apln*-expressing cells. This paracrine signaling regulates tip cell specific behaviors such as filopodia formation, asymmetric daughter cell size after division, and tip-stalk cell shuffling. Furthermore, by using genetic interaction experiments and transgenic biosensors, we found that Apelin signaling exerts its regulatory effects through the activation of phosphoinositide 3-kinase (PI3K) and ERK signaling pathways.

## RESULTS

### During ISV sprouting, *apelin* is predominantly expressed in a subpopulation of neural progenitor cells in the dorsal neural tube

**Fig. 1.**
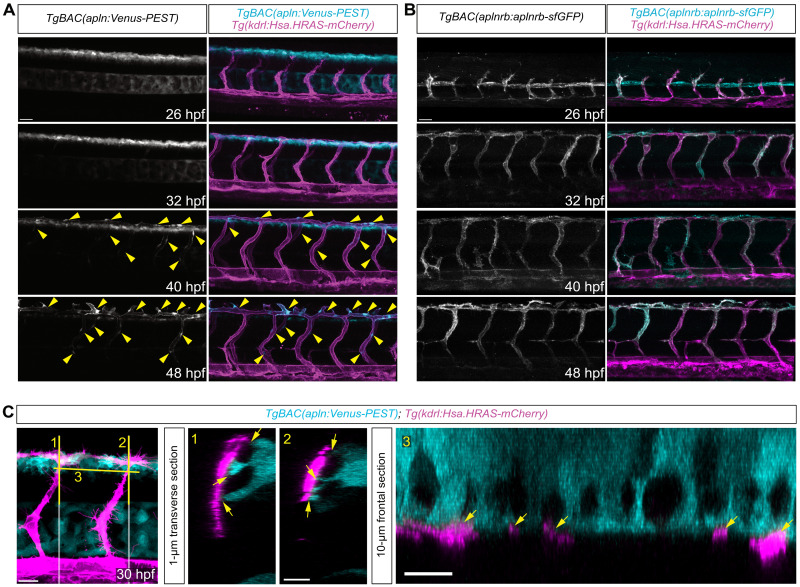
During sprouting of the ISVs, *apln* is expressed in a subpopulation of cells in the dorsal neural tube. (**A**) Still images taken from a time-lapse video of *TgBAC(apln:Venus-PEST)*; *Tg(kdrl:HsHRAS-mCherry)* embryos. *apln:*Venus-PEST expression is detectable in cells in the neural tube. Arrowheads point toward *apln:*Venus-PEST expression in ECs. (**B**) Confocal projections of *Tg(aplnrb:aplnrb-tagRFP-sfGFP)*; *Tg(kdrl:HsHRAS-mCherry)* embryos at indicated time points. *aplnrb*:aplnrb-tagRFP-sfGFP expression is detectable in ECs. (**C**) Confocal projections of *TgBAC(apln:Venus-PEST)*; *Tg(kdrl:HsHRAS-mCherry)*. Tip cells contact *apln*:Venus-PEST–expressing neural cells (arrows). Yellow lines indicate transverse (1 and 2) or frontal sections (3). Scale bars: 30 μm [(A) and (B)], 20 μm (C), and 10 μm [magnifications 1 to 3 in (C)].

To visualize *apln* expression at a single-cell resolution, we previously generated a transgenic bacterial artificial chromosome (BAC) reporter driving green fluorescent protein (GFP) expression under the control of the *apln* promoter that allowed us to confirm expression of *apln* in tip cells of ISVs (*11*). However, the long half-life of GFP (~26 hours) (*22*) does not allow to monitor transcriptional changes over time. To accurately monitor transcriptional changes over time, we developed a new reporter and replaced GFP with Venus-PEST, which has a shorter half-life (*23*). Confocal time-lapse imaging was performed on *TgBAC(apln:Venus-PEST)*; *Tg(kdrl:HsHRAS-mCherry)* transgenic zebrafish embryos between 26 and 48 hours after fertilization (hpf) to track *apln:*Venus-PEST expression (Fig. 1A and movie S1). To our surprise, we observed that *apln:*Venus-PEST expression was highest in cells located in the dorsal neural tube at 26 and 32 hpf and its expression in the vasculature became apparent only after the formation of the DLAV at 40 hpf. Notably, we found that the tip cells extended dorsally toward the *apln:*Venus-PEST–expressing cells at 26 hpf (Fig. 1A). Once the tip cells reach the dorsal side of the embryo, they begin to form a T-shape, connect, and establish the DLAV (*24*). We observed that tip cells migrate along *apln:*Venus-PEST–expressing cells and maintained close contact (Fig. 1, A and C, and fig. S1A). At 48 hpf, *apln*:Venus-PEST expression was strongest in the ECs of the DLAV, while *apln*:Venus-PEST expression in the neural tube decreased (Fig. 1A and fig. S1A). We found that the novel *TgBAC(*apln*:Venus-PEST)* reporter (Fig. 1A and fig. S1, A and B) reflects endogenous *apln* expression, as determined by in situ hybridization (fig. S1, B and C). However, while we could not detect *apln*:Venus-PEST expression in tip cells before 32 hpf, faint *apln* mRNA expression in tip cells was detectable by in situ hybridization at 24 hpf (Fig. 1A and fig. S1C). This discrepancy between the endogenous expression and the reporter expression could be due to the delay between the presence of the mRNA and folding of the fluorophore. In addition, we detected *aplnrb* expression predominantly in the vasculature between 24 and 48 hpf (Fig. 1B and fig. S1C).

To analyze if the *apln*-expressing cells may be neural crest cells or oligodendrocyte precursors, we imaged triple *TgBAC(apln:Venus-PEST)*; *Tg(sox10:GAL4-VP16); Tg(UAS-E1B:NTR-mCherry)* transgenic embryos. However, we could not detect any coexpression of *apln:*Venus-PEST *with sox10:*GAL4-VP16*; UAS-E1B:*NTR-mCherry expression (fig. S2A). Next, we investigated if the *apln*-expressing cells in the dorsal neural tube represent differentiated neurons. Therefore, we genetically labeled neurons and *apln*-expressing cells by using the [*Tg(elavl3:GAL4-VP16); Tg(UAS-E1B:NTR-mCherry)*] and *TgBAC(apln:Venus-PEST)* reporter lines. While we observed that most *apln:*Venus-PEST–expressing cells did not colocalize with *elavl3:*GAL4-VP16*; UAS-E1B:*NTR-mCherry-expressing neurons, we could observe few double-positive cells that localized ventrally to the large cell bodies of Rohon-Beard neurons (fig. S2B, arrowheads).

**Fig. 2.**
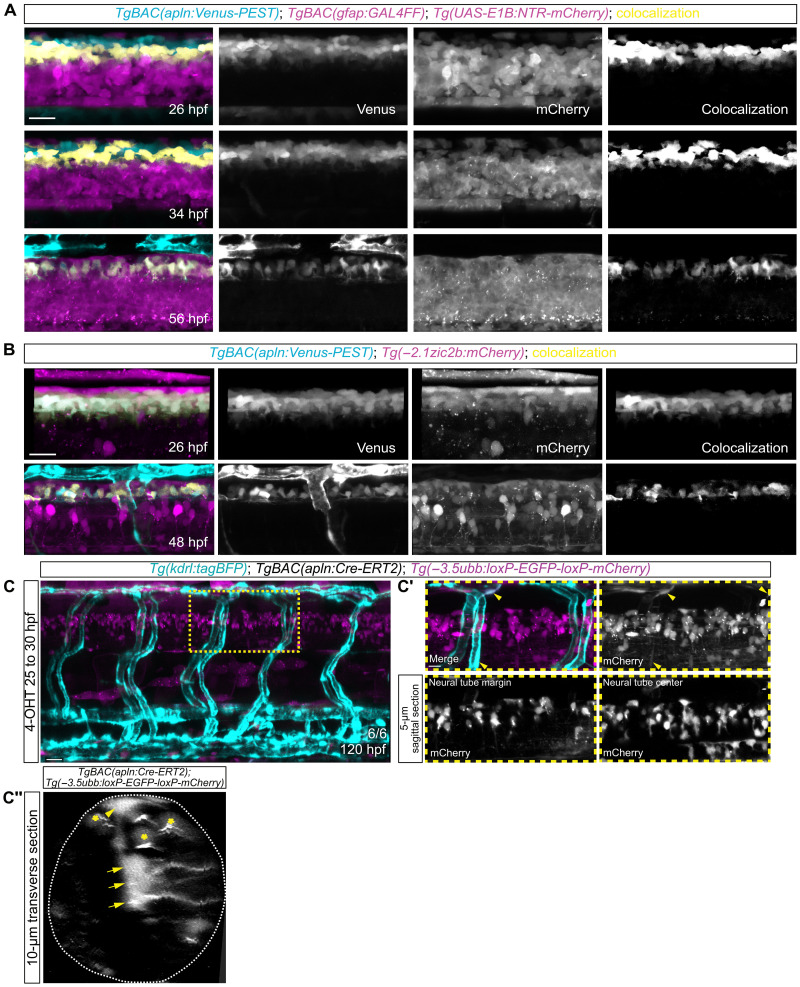
During sprouting of the ISVs, *apln* is expressed in dorsal neural progenitors in the neural tube. (**A**) Confocal projections of *TgBAC(gfap:GAL4FF); Tg(UAS-E1B:NTR-mCherry)*; *TgBAC(apln:Venus-PEST)* transgenic embryos. *apln:*Venus-PEST expression is detectable in *gfap*:GAL4FF*; UAS-E1B*:NTR-mCherry–expressing cells (colocalization channel). Only *gfap:*GAL4FF–expressing cells retain *apln*:Venus-PEST signal until 56 hpf. (**B**) Confocal projections of *TgBAC(apln:Venus-PEST)*; *Tg(−2.1zic2b:mCherry)* double transgenic embryos. *apln:*Venus-PEST expression is detectable in *zic2b*:mCherry–expressing cells (colocalization channel). (**C**) Confocal projection of a *Tg(kdrl:TagBFP); TgBAC(apln:Cre-ERT2); Tg(−3.5ubb:loxP-EGFP-loxP-mCherry)* transgenic embryo at 120 hpf. Recombination was induced from 25 to 30 hpf by adding 4-OHT. Regions for magnification are marked by the yellow dashed box. *N* = 6 embryos. (**C′**) Arrowheads point toward mCherry-expressing ECs. Note the positive neurons in the margin and the positive progenitors in the center of the neural tube. (**C″**) Transverse section of the neural tube of a *TgBAC(apln:Cre-ERT2); Tg(−3.5ubb:loxP-EGFP-loxP-mCherry)* embryo at 120 hpf. Arrows point to projections of radial glia cells; arrowhead marks ventrally extending projection of radial glia-like roof plate cells; asterisks mark neurons. Scale bars: 30 μm [(A) and (B)], 20 μm (C), and 10 μm [(C′) and (C″)].

Considering that radial glial cells represent a substantial proportion of cells in the developing zebrafish neural tube (*25*), we investigated the expression of *apln* in radial glia cells by using the [*TgBAC(gfap:GAL4FF)*; *Tg(UAS-E1B:NTR-mCherry)*] reporter line (Fig. 2A). We observed coexpression of *apln:*Venus-PEST with *gfap:*GAL4FF; *UAS-E1B:*NTR-mCherry at 26 hpf, and at 56 hpf, all cells positive for Venus-PEST in the neural tube were positive for the mCherry (Fig. 2A). To further characterize the cell population in the neural tube that express *apln*, we analyzed optical transverse sections (fig. S3A). We observed *apln*-expressing cells in a cell population limited to the dorsal part of the neural tube (fig. S3, A and B) and that most of the cells exhibit characteristics of (radial) glial cells (fig. S3C), while fewer cells express neuronal markers (fig. S2B). Therefore, we hypothesized that these *apln*-expressing cells could be progenitor cells, capable of developing into different cell types. To test this hypothesis, we assessed if the *apln*^+^ cells express the well-established neural progenitor cell marker Nestin (Nes) (*26*). Upon injecting of a plasmid expressing mScarlet-I3 under the control of the *nes* promoter, we could detect double *apln*^+^ *nes*^+^ positive cells at 52 hpf (fig. S4A). However, most cells that strongly express *nes*:mScarlet-I3 localize ventrally to the *apln:*Venus-PEST–expressing cells, which is in line to what we observed in the *TgBAC(nes:EGFP)* transgenic line (fig. S4, A and B). Because we observed that the *apln*-expressing cells are located in the dorsal part of the neural tube, we analyzed if these cells are part of the roof plate. To test this, we analyzed the expression of *zic2b*, a well-known marker for roof plate cells, as well as dorsal progenitors, interneurons, and cranial neural crest domains (*27*–*31*). Therefore, we generated two novel transgenic reporter lines for zic2b [*Tg(zic2b:mCherry)*, *Tg(zic2b:GAL4-VP16)*]. By analyzing *Tg(zic2b:mCherry)*; *TgBAC(apln:Venus-PEST)* double transgenic embryos, we could detect *zic2b:*mCherry expression in the dorsal neural tube at 26, 32, and 48 hpf (Fig. 2B). Notably, we observed that the *apln:*Venus-PEST*^+^* cells express *zic2b:*mCherry and localize ventrally to the cells that displayed the highest *zic2b:*mCherry expression (Fig. 2B). Further analysis revealed that the *Tg(zic2b:mCherry)* reporter marks interneurons (fig. S5A, neural tube margin) and roof plate cells (fig. S5, A to C, central neural tube) (*28*, *32*). We observed axonal-like projections emerging from cells expressing *apln:*Venus-PEST and *elavl3:*GAL4-VP16*; UAS-E1B:*NTR-mCherry or *zic2b:*GAL4-VP16*; UAS-E1B:*NTR-mCherry (fig. S6, A to D, arrows). Notably, the expression of *apln:*Venus-PEST in these cells rapidly declines once the projection is formed (fig. S6, A and B, arrows). Considering their position within the neural tube (figs. S2 to S5), their distinct morphology, characterized by central cell bodies with projections extending outward of the *apln:*Venus-PEST–expressing cells (fig. S3C), as well as their expression of *zic2b* (fig. S5, A and B), we propose that these cells are dorsal neural progenitor cells, which can give rise to interneurons. To trace the fate of the *apln*-expressing cells over time, we performed inducible genetic lineage tracing using *TgBAC(*apln*:Cre-ERT2)*; *Tg(−3.5ubb:loxP-EGFP-loxP-mCherry)*; *Tg(kdrl:TagBFP)* transgenic embryos and induced recombination from 25 to 30 hpf by adding 4-Hydroxytamoxifen (4-OHT) to water (Fig. 2C). Upon 4-OHT treatment, *apln:Cre-ERT2*–expressing cells and their descendants are permanently labeled with mCherry. At 120 hpf, we identified mCherry-labeled cells in the dorsal half of the neural tube as well as mCherry-labeled ECs (Fig. 2, C and C′, arrowheads). Among the mCherry-labeled cells, we identified cell types with the characteristic morphology of neurons (Fig. 2C″, asterisks), radial glial cells (Fig. 2C″, arrows), and roof plate progenitor cells (Fig. 2C″, arrowheads, and movie S2), confirming our earlier observations. In conclusion, our data indicate that *apln* is transiently expressed (24 to 56 hpf) in dorsal neural progenitor cells in the zebrafish neural tube. After 32 hpf, *apln* expression in dorsal neural progenitor cells declines, likely caused by the differentiation of the progenitor cells. Simultaneously, vascular *apln* expression in the DLAV increases from 32 hpf onward.

### Neural progenitor cell–derived Apelin regulates tip cell migration and DLAV formation

**Fig. 3.**
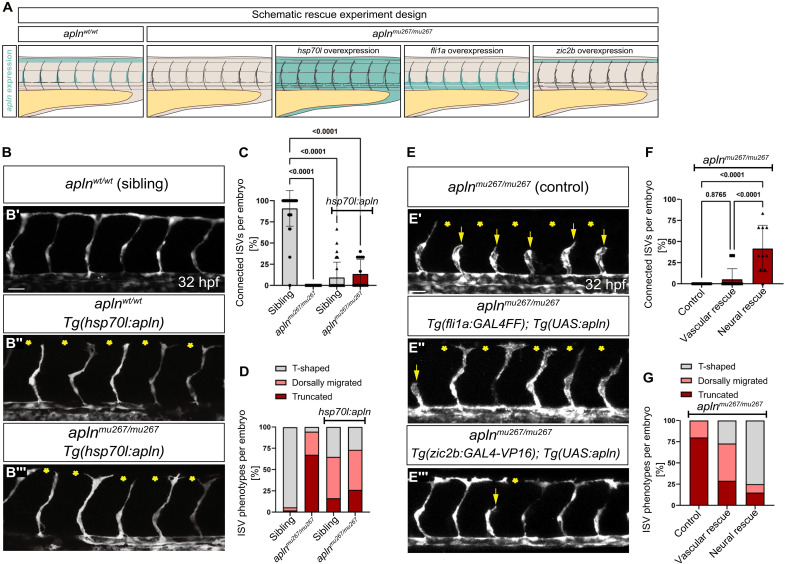
Neural progenitor cell–derived Apelin drives tip cell elongation and DLAV formation. (**A**) Schematic model of the tissue-specific *apln* expression rescue experiment. (**B** and **E**) Confocal projections of *Tg(kdrl:gfp)* (B) or *Tg(fli1a:EGFP)* (E) embryos. (B) *apln* overexpression in *Tg(hsp70l:apln)* leads to defects in the formation of the DLAV (asterisks) and is unable to rescue DLAV formation in *apln* mutants (asterisks) at 32 hpf. The heat shock was performed at 24 hpf. (**C**) Percentage of ISVs that formed at least one DLAV connection. *P* values calculated by ANOVA with Kruskal-Wallis test. (**D**) Percentage of ISVs displaying one of the three indicated vessel phenotypes. *P* values calculated by ANOVA with Tukey’s test can be found in fig. S7I. [(C) and (D)] Sibling control, *N* = 35 embryos; *apln^mu267/mu2677^* control, *N* = 15 embryos; sibling *hsp70l:apln*, *N* = 33 embryos; *apln^mu267/mu2677^ hsp70l:apln*, *N* = 14 embryos; six ISVs per embryo from four experiments (E). Vascular expression of *apln*, by using *Tg(fli1a:GAL4FF); Tg(UAS:LIFEACT-EGFP)*; *Tg(UAS:apln)* embryos, only partially rescues the dorsal migration of tip cells but not DLAV formation (asterisks) in *apln* mutants. Neural expression of *apln*, by using *Tg(zic2b:GAL4-VP16)*; *Tg(UAS:apln)* embryos, rescues the dorsal migration of tip cells and DLAV formation (asterisks) in *apln* mutants. (**F**) Percentage of ISVs that formed at least one DLAV connection. *P* values calculated by ANOVA with Kruskal-Wallis test. (**G**) Percentage of ISVs displaying one of the three indicated vessel phenotypes. *P* values calculated by ANOVA with Tukey’s test can be found in fig. S7I. [(F) and (G)] Control, *N* = 19 embryos; neural overexpression, *N* = 10 embryos; vascular overexpression, *N* = 19 embryos; six ISVs per embryo from ≥2 experiments. Scale bars: 30 μm.

*apln* is known to be expressed in vascular tip cells (*11*, *20*). Our discovery that *apln* is expressed in dorsal neural progenitor cells during ISV sprouting led us to investigate the respective contributions of neural and vascular Apln in the regulation of sprouting angiogenesis (Fig. 3A). First, we tested if Apln gradients attract tip cells toward the neural tube. To address this question, we used a heat shock–inducible transgenic line [*Tg(hsp70l:apln)*] to ubiquitously overexpress *apln*, potentially disrupting local Apelin gradients. A single heat shock at 24 hpf resulted in a loss of DLAV formation at 32 hpf in most *Tg(hsp70l:apln)* positive embryos (9% connected ISVs) (Fig. 3, B and C, and fig. S7A). Although the ubiquitous overexpression of *apln* failed to restore DLAV formation in *apln* mutants, *apln* mutants with ubiquitous overexpression of *apln* displayed a significant reduction in the number of short/truncated ISVs (*apln* mutant with 67% truncated ISVs and *apln* mutant with ubiquitous overexpression 26% truncated ISVs) (Fig. 3, B‴, C, and D). To selectively restore *apln* expression in *apln* mutants in a tissue-specific manner, we used the GAL4-UAS (Upstream Activating Sequence) system and generated a new *Tg(UAS:apln)* line. Overexpression of *apln* in the vasculature of *apln* mutant embryos by using *Tg(fli1a:GAL4FF); Tg(UAS:apln)* embryos failed to rescue DLAV formation (5% connected ISVs) (Fig. 3, E″ and F) but reduced the number of truncated ISVs [*apln* mutant (control) with 80% truncated ISVs and vascular overexpression with 29% truncated ISVs] (Fig. 3G). To investigate if *apln* expression in the dorsal neural tube could rescue the *apln* mutant phenotype, we analyzed *Tg(zic2b:GAL4-VP16); Tg(UAS:apln)* embryos. Notably, overexpression of *apln* in *zic2b*-expressing cells rescues DLAV formation in 42% of ISVs and restores T-shape formation in 75% of ISVs in *apln* mutants (Fig. 3, E‴, F, and G). Furthermore, *zic2b*-mediated *apln* expression reduced the number of truncated ISVs to an even greater extent than vascular overexpression (15% truncated ISVs). We next examined if Apelin is required in *zic2b*-expressing cells or if the location in the neural tube is required for angiogenesis of the ISVs. To this aim, we overexpresses *apln* in neurons using the *Tg(elavl3:GAL4-VP16)* transgenic line. Overexpression of *apln* in neurons leads to a rescue of DLAV formation in 62% of ISVs, as well as T-shape forming vessels by 32 hpf (fig. S7, B to F). Because we observed *apln:*Venus-PEST expression in ECs at 48 hpf, we analyzed a potential role of vascular-derived *apln* at 48 hpf (fig. S7, G and H). Consistent with our data at 32 hpf, we found that, even at 48 hpf, vascular-derived *apln* does not rescue DLAV formation (37% connected ISVs) to the same extent as neural progenitor cell–derived *apln*.

Overall, our data show that tip cells in the ISV require a source of Apelin from the neural tube to form the T-shape and anastomose to form the DLAV. In contrast, dorsal migration of tip cells toward the neural tube is less dependent on Apelin from the neural tube.

### Apelin signaling drives the elongation of tip cells

**Fig. 4.**
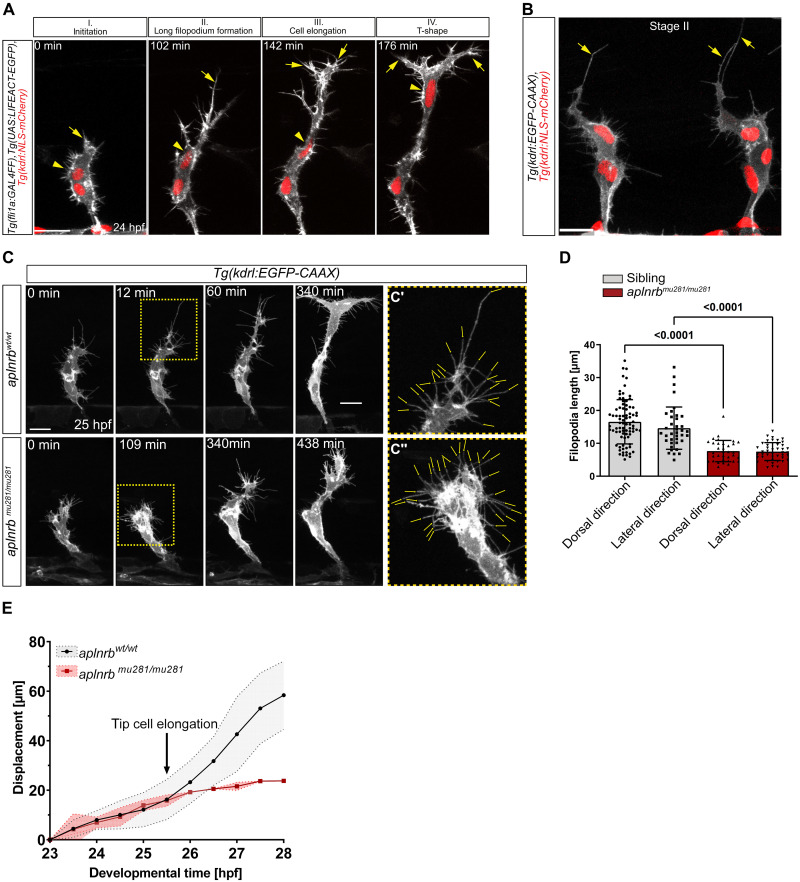
Apelin signaling drives endothelial tip cell elongation. (**A** to **C**) Confocal maximum intensity projections of sprouting ISVs. (A) Still images taken from a time-lapse video of *Tg(fli1a:GAL4FF); Tg(UAS:LIFEACT-EGFP); Tg(kdrl:NLS-mCherry)* embryos. During sprouting, the tip cells exhibit four characteristic morphological stages (I to IV). Arrows point toward the leading edge/dominant filopodium; arrowheads mark the nucleus of the tip cell. (B) High-resolution image of stage II of an embryo expressing *Tg(kdrl:EGFP-CAAX)* and *Tg(kdrl:NLS-mCherry)*. Arrows point toward long filopodia. (C) Still images of time-lapse videos comparing sprouting of tip cells in wild-type and *aplnrb^mu281/mu281^* ISVs expressing *Tg(kdrl:EGFP-CAAX).* Regions for magnification are marked by the yellow dashed box. (**C′** and **C″**) Arrows point to filopodia. (**D**) Filopodia length in *aplnrb^mu281/mu281^* in comparison to siblings in dorsal (45° to 90° relative to the DA) and lateral (0° to 45° relative to the DA) directions. Siblings, *n* = 111 filopodia from *N* = 13 embryos; *aplnrb^mu281/mu281^*, *n* = 39 filopodia from *N* = 9 embryos. (**E**) Dorsal displacement of tip cells from 23 to 28 hpf in wild-type and *aplnrb^mu281/mu281^* embryos. Wild-type, *n* = 10 ISVs; *aplnrb^mu281/mu281^*, *n* = 7 ISVs. Outline is the SD. Scale bars: 20 μm [(A) and (C)] and 10 μm [(B), (C′), and (C″)].

On the basis of our findings, we aimed to characterize the impact of neural progenitor cell–derived *apln* on tip cell behavior and performed high-resolution time-lapse imaging of the actin cytoskeleton of tip cells by analyzing *Tg(fli1a:GAL4FF)*; *Tg(UAS:LIFEACT-EGFP); Tg(kdrl:NLS-mCherry)* embryos (Fig. 4A). We found that tip cells exhibit four distinct morphological changes during ISV sprouting (Fig. 4A): (i) Initially, tip cells exhibit a compact morphology; (ii) formation of a long dorsal filopodium after crossing the horizontal myoseptum; (iii) elongation of the cell body while the nucleus remained stationary; and (iv) formation of T-shaped connections with neighboring ISV tip cells, accompanied by dorsal movement of the nucleus.

To improve visualization of dynamic filopodia formation, we generated a novel transgenic line [*Tg(kdrl:EGFP-CAAX)*] expressing membrane-bound GFP under the control of the *kdrl* promoter, providing a higher spatial resolution of membrane dynamics (Fig. 4, B and C). Consistent with previous findings, we observed that tip cells formed one or two dominant filopodia (*3*), which preceded tip cell elongation (Fig. 4B). To investigate the role of Apelin signaling on tip cell behavior at a single-cell resolution, we analyzed *aplnrb* mutant embryos from 25 hpf onward (Fig. 4C and movie S3). The initial phenotype of *aplnrb* mutant tip cells is not markedly different from that of wild-type tip cells, although migration was slower in *aplnrb* mutant tip cells (Fig. 4C, 0 min). However, as wild-type tip cells reached the dorsal edge of the notochord/horizontal myoseptum and start to elongate (Fig. 4, C and E), *aplnrb* mutant tip cells fail to form a dominant long filopodium (Fig. 4C) and did not elongate (Fig. 4E). By analyzing filopodia formation, we observed that filopodia from control embryos are longer when extending toward the dorsal side [45° to 90° relative to the dorsal aorta (DA)] than toward the lateral side (0° to 45° relative to the DA) (mean dorsal, 16.54 μm; lateral, 14.61 μm) (Fig. 4D). However, *aplnrb* mutant embryos exhibited a reduction in filopodia length in both dorsal and lateral directions (mean dorsal, 7.65 μm; lateral, 7.48 μm) (Fig. 4D). The lengths of filopodia in *aplnrb* mutants is nearly identical in both directions, and filopodia longer than 20 μm are completely absent (Fig. 4D). To investigate if Apelin signaling modulates actin dynamics in tip cells, we treated embryos with latrunculin B to block actin polymerization (*33*). Treatment with 375 nM latrunculin B does not interfere with embryonic development but impairs filopodia formation in ISVs, resulting in slower directional migration and failure to form the DLAV (*3*), phenocopying the migratory behavior of *aplnrb* mutant embryos (fig. S8, A and B). Notably, even a lower dose of latrunculin B (125 nM) affected *aplnrb* heterozygous embryos more severely than wild-type siblings (fig. S8, C and D), suggesting a regulatory role of the Apelin pathway in actin dynamics.

**Fig. 5.**
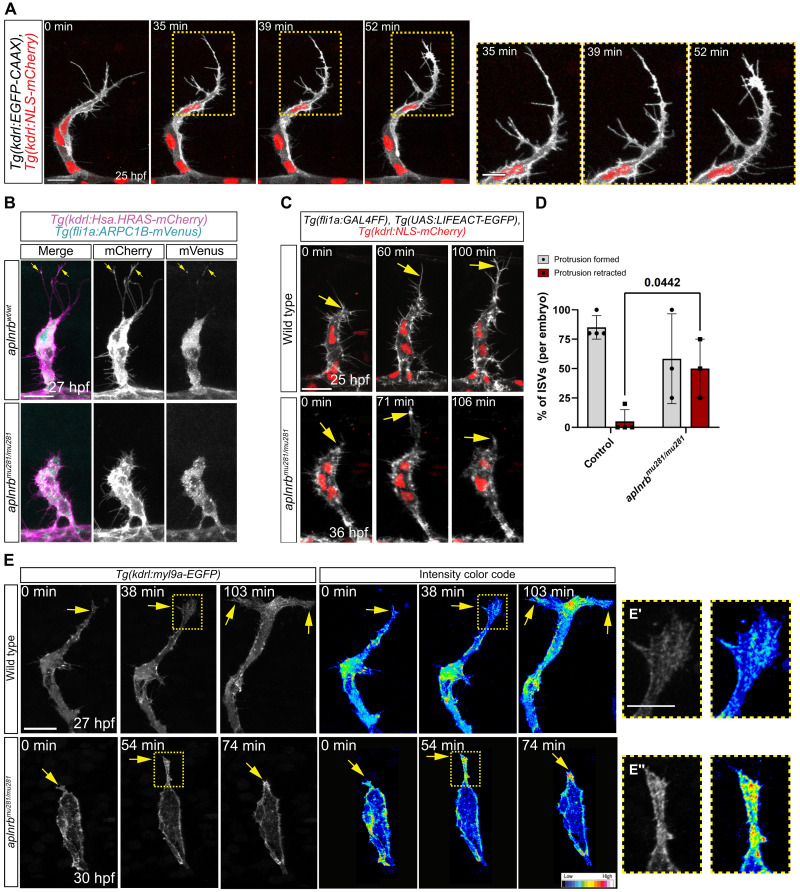
Tip cells elongate by the transformation of the long filopodia into the cell body. (**A**) Still images taken from a time-lapse video of a sprouting ISV expressing *Tg(kdrl:EGFP-CAAX); Tg(kdrl:NLS-mCherry)* depicting tip cell elongation by filopodia widening. Regions for magnification are marked by the yellow dashed box. *N* = 3 embryos. (**B**) Confocal projections of *aplnrb^wt/wt^* or *aplnrb^mu281/mu281^* embryos expressing *Tg(fli1a:ARPC1B-mVenus)* and *Tg(kdrl:Hsa.HRAS-mCherry)*. ARPC1B-mVenus localization in dominant long filopodia (arrows). *aplnrb^wt/wt^*, *N* = 4 embryos; *aplnrb^mu281/mu281^*, *N* = 4 embryos. (**C**) Still images taken from a time-lapse video of a sprouting tip cells expressing *Tg(fli1a:GAL4FF)*; *Tg(UAS:LIFEACT-EGFP*; *Tg(kdrl:NLS-mCherry)*. Arrows point toward protrusions. Protrusions of tip cells in *aplnrb* mutants retract. (**D**) Quantification of protrusions formed and retracted by tip cells in embryos from confocal time-lapse movies recorded between 23 and 30 hpf (control) or 30 and 39 hpf (*aplnrb^mu281/mu281^*). Control, *N* = 4 embryos; *aplnrb^mu281/mu281^*, *N* = 3 embryos; four ISVs per embryo. (**E**) Still images taken from a time-lapse video of *Tg(kdrl:myl9a-EGFP)* embryos in wild-type and *aplnrb^mu281/mu281^* embryos. Arrows point toward the protrusions of the tip cell. *aplnrb^mu281/mu281^* embryos exhibit a high myl9a-EGFP localization in retracting protrusions. Wild-type, *n* = 8 ISVs of *N* = 4 embryos; *aplnrb^mu281/mu281^*, *n* = 9 ISVs of *N* = 3 embryos. Regions for magnification are marked by the yellow dashed box. Scale bars: 20 μm [(A), (B), (C), and (E)], 10 μm [magnification in (E′) and (E″)], and 5 μm [magnification in (A)].

Because we found that Apelin signaling is specifically required for the formation of the long filopodia, we next investigated the nature of these filopodia. Notably, we found that tip cells elongated through widening of the dominant filopodium by membrane ruffling (Fig. 5A and movie S4). These observations are reminiscent of the long, Arp2/3 complex-dependent lamellipodium-like protrusions (LLPs) observed in mice (*34*). To understand the transition from a dominant filopodium to cell elongation, we generated a transgenic line that expresses an ARPC1B-mVenus fusion protein in ECs (Fig. 5B and fig. S9, A and B). Validation of ARPC1B-mVenus localization matched published data on the Arp2/3 complex, demonstrating ARPC1B-mVenus localization in lamellipodia and cell-cell contacts (fig. S9A). Time-lapse imaging revealed Arp2/3 localization to long filopodia in tip cells, followed by membrane ruffling (fig. S9B, arrows, and movie S5), but not to shorter, transient filopodia (fig. S9B, arrowheads, and movie S5). These findings indicate the transition of long filopodia into LLPs before cell elongation. Because *aplnrb* mutants do not form long filopodia, we were not able to detect if Apelin signaling directly controls Arp2/3 localization in long filopodia (Fig. 5B). To analyze the function of the Arp2/3 complex during tip cell elongation, we treated embryos with the Arp2/3 inhibitor CK666 at 23 hpf, shortly before the initiation of cell elongation (fig. S9, C and D). We found that embryos treated with CK666 exhibited truncated ISVs, confirming the essential role of Arp2/3 in tip cell elongation.

To gain deeper insights into the phenotype of *aplnrb* mutant embryos, we analyzed time-lapse videos of sprouting ISVs in *Tg(fli1a:GAL4FF)*; *Tg(UAS:LIFEACT-EGFP)*; *Tg(kdrl:NLS-mCherry)*embryos. In *aplnrb* mutants, we observed protrusions—albeit at a later time point. However, these protrusions retracted (Fig. 5, C and D). To determine if tip cells actively retract protrusions, we imaged tip cells in embryos expressing GFP–myosin II in the vasculature [*Tg(kdrl:myl9a-EGFP)*]. Elongating tip cells of wild-type embryos showed myosin localization at the base of filopodia and the cell cortex (Fig. 5, E and E′). Conversely, *aplnrb* mutant embryos exhibited high myosin enrichment in protrusions during retraction (Fig. 5, E and E″; fig. S10, A to C; and movie S6).

In summary, our results show that Apelin signaling in tip cells is required for the formation of long filopodia that widen to allow rapid cell elongation. This might, in turn, require Arp2/3-dependent actin polymerization. In addition, loss of Apelin signaling leads to mislocalization of myosin to the dorsal protrusions, leading to filopodia retraction by actomyosin contraction.

### Apelin signaling induces tip-stalk cell asymmetry

**Fig. 6.**
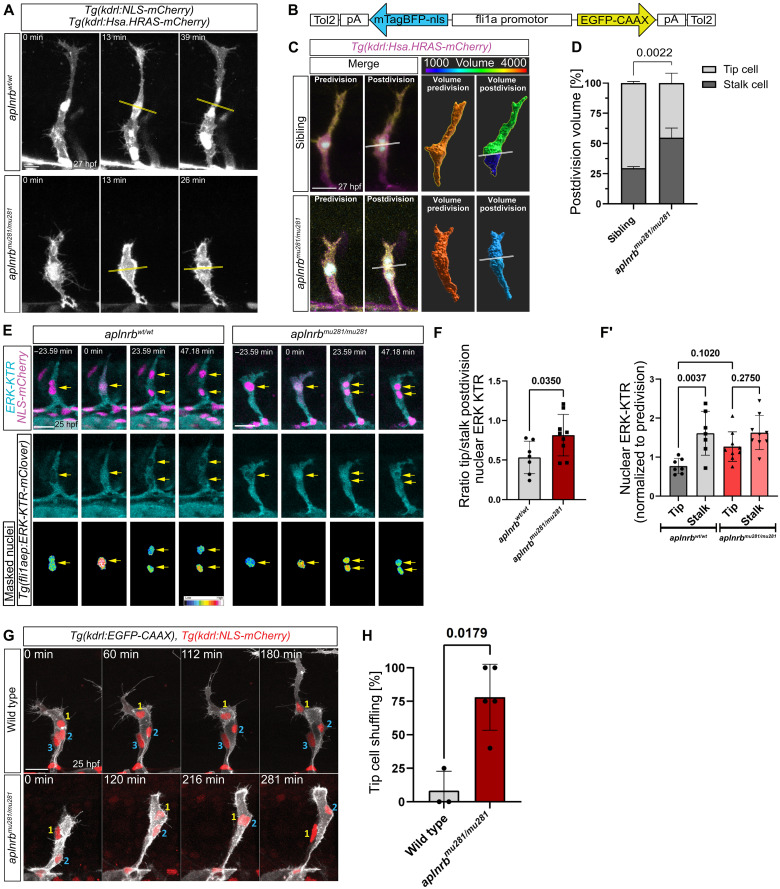
Apelin signaling induces tip-stalk cell asymmetry. (**A**) Tip cell division in *aplnrb^wt/wt^* and *aplnrb^mu281/mu281^; Tg(kdrl:NLS-mCherry); Tg(kdrl:HsHRAS-mCherry)* embryos. (**B**) Plasmid design. (**C**) Single labeled tip cells in siblings and *aplnrb^mu281/mu281^* embryos. Color-coded for cell volume. (**D**) Postdivision cell volumes of daughter tip and stalk cells. Siblings, *n* = 3 tip cells of *N* = 3 embryos; *aplnrb^mu281/mu281^*, *n* = 4 tip cells of *N* = 4 embryos; *P* value calculated by two-tailed *t* test. (**E**) Still images of a time-lapse video of *Tg(fli1aep:ERK-KTR-Clover); Tg(kdrl:NLS-mCherry)* embryos, comparing ERK activity in tip cells of *aplnrb^wt/wt^* and *aplnrb^mu281/mu281^* embryos during cell division. Arrows point to nuclei. Color-coded for nuclear intensity. (**F**) Nuclear ERK-KTR intensity during tip cell divisions. *n* = 7 tip cell divisions of *N* = 4 embryos; *aplnrb^mu281/mu281^*, *n* = 9 tip cell divisions of *N* = 6 embryos. (F) Relative postdivision intensity of daughter tip/stalk cells. *P* value calculated by two-tailed *t* test. (**F′**) Absolute nuclear ERK-KTR intensities postdivision normalized to predivision intensity. *P* values calculated by ANOVA with Tukey’s test. (**G**) Still images taken from a time-lapse video of sprouting ISVs from wild-type or *aplnrb^mu281/mu281^* embryos expressing *Tg(kdrl:EGFP-CAAX); Tg(kdrl:NLS-mCherry)*. Numbers indicate initial cell nuclei order (1: tip cell nucleus). (**H**) Frequency that the tip cell is overtaken by a stalk cell at least once during ISV sprouting. Siblings, *n* = 3 embryos; *aplnrb^mu281/mu281^*, *n* = 6 embryos; four to six ISVs per embryo. *P* value calculated by Mann-Whitney test. The division plane is marked by white line [(A) and (C)]. Scale bars: 20 μm [(C), (E), and (H)] and 10 μm (A).

Tip cells undergo asymmetric divisions during their migration, which maintains a larger tip cell size and high VEGF signaling after division (*10*). By analyzing time-lapse videos of tip cell divisions in *Tg(kdrl:Hsa.HRAS-mCherry); Tg(kdrl:NLS-mCherry)* double transgenic embryos, we observed a cell size asymmetry after cell division of control tip cells (Fig. 6A). Notably, tip cells in *aplnrb* mutant embryos underwent symmetrical cell division (Fig. 6A). To quantify the volume of tip cells, we used a mosaic labeling strategy and labeled single tip cells by nuclear mTagBFP-nls and membrane-localized EGFP-CAAX (Fig. 6B). We found that the volume of nondividing tip cells in both wild-type and *aplnrb* mutant embryos increased over time (fig. S11, A and B). However, the volume of tip cells in *aplnrb* mutant embryos was smaller on average at both 25 and 30 hpf (fig. S11, A and B). Using our mosaic labeling strategy, we further investigated the relative volume of daughter cells after tip cell division between 25 and 30 hpf. Consistent with a previous report (*10*), tip cells in wild-type embryos exhibited an asymmetric daughter cell volume after division, resulting in larger daughter tip cells (Fig. 6, C and D). In contrast, division of tip cells in *aplnrb* mutants caused daughter cells of even size, and occasionally, the daughter tip cells were even smaller than the daughter stalk cells (Fig. 6, C and D). Asymmetric tip cell division leads to asymmetric partitioning of mRNA, such as *kdrl* mRNA, and higher ERK activity in daughter tip cells compared to daughter stalk cells (*10*, *35*). To determine if the symmetric division of tip cells in *aplnrb* mutant embryos affect ERK activity, we analyzed cell divisions using confocal time-lapse videos of the transgenic ERK activity reporter *Tg(fli1aep:ERK-kinase translocation reporter (KTR)-Clover)*, which has been used to assess the dynamic Erk activity in ECs (Fig. 6, E and F) (*35*). The ERK-KTR-Clover is a synthetic reporter that enables quantification of Erk phosphorylation events in cells based on the nucleocytoplasmic ratio of the fluorescent fusion protein (*36*). After phosphorylation by ERK, the reporter gets exported from the nucleus, meaning that a lower nuclear signal equals higher ERK activity. In line with previous findings (*35*), we observed asymmetric ERK activity after cell division in daughter tip cells and daughter stalk cells in control embryos (Fig. 6, E and F′). In contrast, we observed similar ERK activity after cell division between daughter tip and daughter stalk cells in *aplnrb* mutants (Fig. 6, E and F′), suggesting that higher ERK activity in daughter tip cells depends on the cell size asymmetry regulated by Apelin signaling. Considering that tip and stalk cells dynamically compete for the tip cell position during angiogenic sprouting (*2*), we investigated if Apelin signaling maintains the migratory advantage of tip cells. We observed that loss of *aplnrb* lead to a dynamic shuffling of tip and stalk cells, a phenotype rarely observed in wild-type embryos (Fig. 6, G and H). We hypothesize that the shuffling of tip cells and stalk cells in *aplnrb* mutants might be promoted by the symmetric cell divisions. However, we also observed dynamic tip cell shuffling without tip cell divisions (Fig. 6G and movie S7).

These results suggest that the Apelin signaling pathway is an instructive signal for asymmetric daughter cell size after tip cell divisions. As a result, ERK activity in tip cells remains higher than in stalk cells even during cell division, thereby provides tip cells with the migratory advantages necessary to prevent tip-stalk cell shuffling.

### Apelin signaling drives sprouting via PI3K and ERK signaling in vivo

**Fig. 7.**
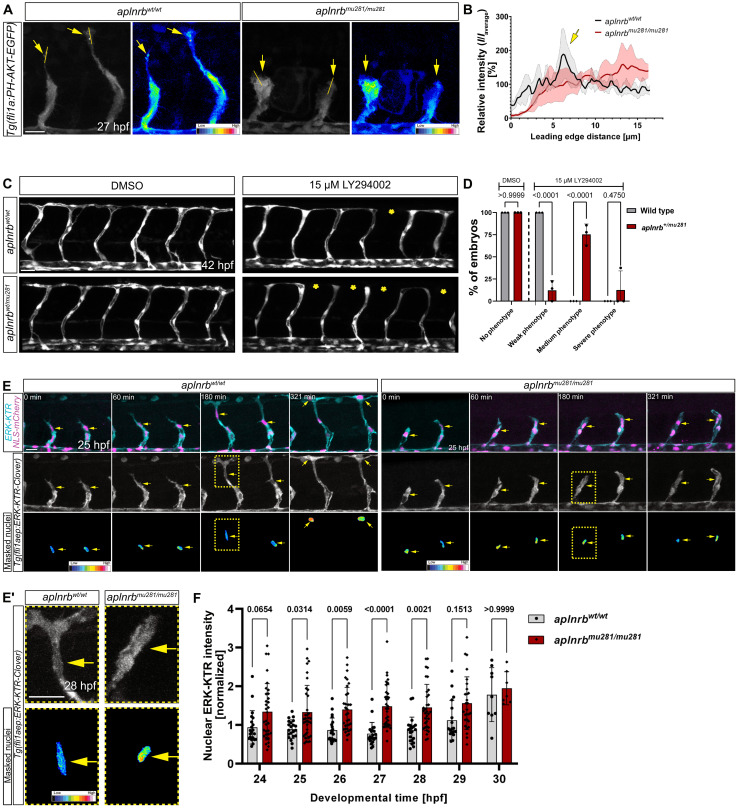
Apelin signaling drives sprouting via PI3K and ERK in vivo. (**A**) Confocal projections of the ISVs of *Tg(fli1a:PH-AKT-EGFP) aplnrb^wt/wt^* or *aplnrb^mu281/mu281^* embryos. Yellow lines indicate regions for line graph measurements (B), and arrows point toward the cell edge. (**B**) Line graph measurements comparing the PH-AKT-EGFP signal in *aplnrb^wt/wt^* and *aplnrb^mu281/mu281^* embryos. The relative intensity was normalized to the average intensity across the line. Lines display the mean value; the faint outlines the SDs. *aplnrb^wt/wt^*, *n* = 4 tip cells of *N* = 3 embryos; *aplnrb^mu281/mu281^* embryos, *n* = 3 tip cells of *N* = 2 embryos. (**C**) Confocal projections of *Tg(fli1a:EGFP) aplnrb^wt/wt^* and *aplnrb^wt/mu281^* embryos treated from 20 to 42 hpf with 15 μM LY294002 or DMSO. Asterisks mark missing DLAV segments. (**D**) Quantification of the vascular phenotype of (C). DMSO *aplnrb^wt/wt^*, *N* = 15 embryos; *aplnrb^wt/mu281^*, *N* = 24 embryos; *P* values determined by ANOVA with Tukey’s test. (**E**) Still images taken from time-lapse videos of a sprouting ISV of *Tg(fli1aep:ERK-KTR-Clover); Tg(kdrl:NLS-mCherry) aplnrb^wt/wt^* and *aplnrb^mu281/mu281^* embryos. Arrows point to the nuclei of the tip cell. Masked nuclei channel color-coded for intensity. Regions for magnification are marked by the yellow dashed box (**E′**). (**F**) Quantification of nuclear ERK intensity *aplnrb^wt/wt^* mutants and *aplnrb^mu281/mu281^* mutants from 24 to 30 hpf. *aplnrb^wt/wt^*, *n* = 21 tip cells of *N* = 7 embryos; *aplnrb^mu281/mu281^* embryos, *n* = 37 tip cells of *N* = 11 embryos. *P* values determined by ANOVA with Bonferroni correction. Scale bars: 30 μm (C) and 20 μm [(A), (E), and (E′)].

The Aplnr binds the G protein subunit Gαi and activates downstream signaling pathways such as PI3K and ERK (*37*). To determine if Apelin signaling regulates ISV sprouting through these downstream effectors, we used biosensors and pharmacological inhibitors. To visualize PI3K activity in ECs in vivo, we generated a novel transgenic line [*Tg(fli1a:PH-AKT-EGFP)*] expressing the pleckstrin homology (PH) domain of AKT fused to EGFP, which is commonly used to visualize the localization of phosphatidylinositol 3,4,5-trisphosphate (PIP3) (*38*), the product of PI3K in ECs (Fig. 7A and fig. S12, A and B). We observed PH-AKT-EGFP localization at the leading edge of the elongating (fig. S12, A and B, and movie S8) and T-shaped (fig. S12B) tip cells. Intriguingly, *aplnrb* mutants did not display PH-AKT-EGFP localization toward the leading edge; instead, the PH-AKT-EGFP signal was more evenly distributed throughout the cell (Fig. 7, A and B). To investigate a possible role of PI3K downstream of Apelin signaling, we treated *aplnrb* heterozygous embryos from 20 to 42 hpf with a low concentration of the PI3K inhibitor LY294002 that did not cause severe angiogenic defects in wild-type embryos (Fig. 7, C and D). Notably, heterozygous *aplnrb* mutant embryos treated with the PI3K inhibitor displayed a stronger phenotype compared to wild-type siblings (Fig. 7, C and D). Confocal time-lapse imaging revealed that protrusions retract in embryos treated with a higher dose of the PI3K inhibitor (25 μM LY294002), similarly to what we observed in *aplnrb* mutant embryos (fig. S12, C and D).

Because GPCRs regulate the β and γ catalytic subunits of class I PI3K (*pik3cb* and *pik3cg* in zebrafish), we investigated involvement of these subunits in ISV development. We treated embryos from 20 to 42 hpf with a low dose of the PI3Kγ-specific inhibitor AS-605240 that did not cause severe angiogenic defects in wild-type embryos (fig. S13A) and found that, like the global PI3K inhibitor LY294002, treatment with AS-605240 impaired sprouting in *aplnrb* heterozygous embryos more severely than in their wild-type siblings (fig. S13, A and B). To further assess the potential roles of *pik3cg* and *pik3cb*, we used a genetic approach by injecting three crRNAs targeting different exons (*39*, *40*) of each gene into *Tg(fli1a:EGFP)* embryos and analyzed the F0 crispants at 32 hpf (fig. S13, C and D). We found that most control embryos formed the DLAV, while 62.8% of embryos injected with crRNAs targeting *pik3cg* and 47.8% of embryos injected with crRNAs targeting *pik3cb* fail to form the DLAV (fig. S13, C and D), suggesting that *pik3cg* and *pik3cb* may play a role in ISV sprouting.

Given that stimulation of Aplnr-expressing cells with Apelin causes phosphorylation of ERK in vitro (*41*) and considering the crucial role of ERK activity in ISV development (*42*), we analyzed ERK activity during vessel sprouting in *aplnrb* mutants (Fig. 7, E and F). To track dynamic changes in ERK activity in ISVs over time, we recorded time-lapse videos of the ERK kinase translocation reporter *Tg(fli1aep:ERK-KTR-Clover)* (*35*, *36*) from 24 to 30 hpf (before, during, and after cell elongation) in wild-type and *aplnrb* mutant embryos (Fig. 7, E and F). In wild-type embryos, ERK activity was highest during tip cell elongation at 27 hpf (Fig. 7, E, E′, and F). ERK activity was significantly lower in *aplnrb* mutant embryos (Fig. 7, E, E′, and F). At 30 hpf, the tip cells of wild-type embryos reach the dorsal side of the embryo and begin to form the DLAV. Consistent with previous reports, we observed that ERK activity decreased at this developmental time point (*35*) and became comparable to that of *aplnrb* mutant embryos (Fig. 7, E and F). To test if the reduced ERK activity is a direct or an indirect consequence of the cell extension–actin dynamics phenotype of *apln* mutants, we treated *Tg(fli1aep:ERK-KTR-Clover)* embryos with latrunculin B or CK666 from 24 to 27 hpf and measured ERK activity of tip and stalk cells at 27 hpf (fig. S14). We found that inhibiting actin polymerization and cell elongation equalized the ERK activity of tip and stalk cells in both latrunculin B–treated and CK666-treated embryos (fig. S14, A and B). Therefore, the altered cell shape in *aplnrb* mutants may contribute to the reduced ERK activity that we observed in tip cells.

**Fig. 8.**
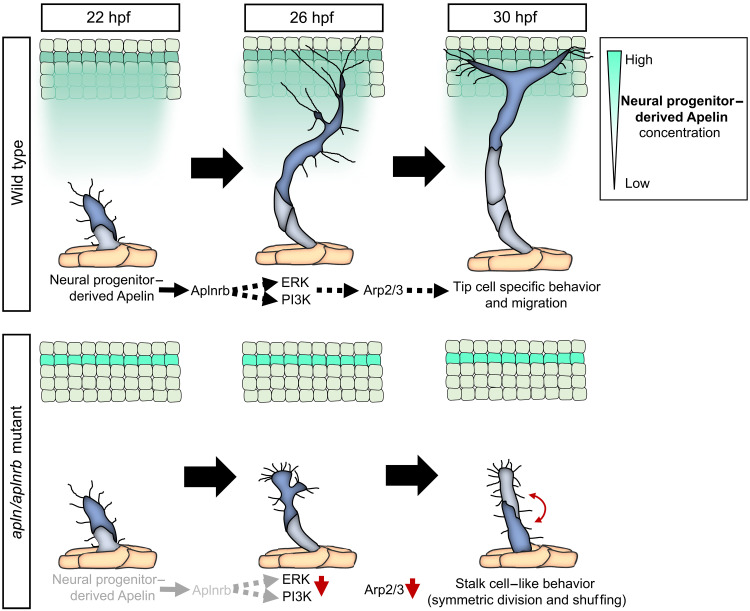
Model of Apelin signaling–mediated neurovascular cross-talk that controls tip cell behavior.

Our findings strongly implicate that PI3K and ERK signaling are downstream effectors of Apelin signaling in sprouting ISVs, and we propose a model by which neurovascular Apelin signaling drives tip cell function by modulating PI3K and ERK activity (Fig. 8).

## DISCUSSION

Previously, we found that Apelin signaling promotes a proangiogenic state in sprouting ECs and is repressed by Notch signaling (*11*). Yet, these findings only partially explain the severe angiogenic defects observed in *apln* and *aplnrb* mutants. Therefore, in this study, we further investigated how Apelin signaling regulates EC dynamics during angiogenic sprouting.

Apelin is known to be highly expressed in tip cells (*11*, *20*), suggesting its potential autocrine function during angiogenesis. However, our findings indicate that paracrine Apelin from neural progenitor cells attracts tip cells during angiogenesis in the zebrafish trunk. Neural progenitor cell–derived Apelin controls the dorsal migration of tip cells and the formation of the DLAV. To form a T-shape and subsequently the DLAV, tip cells might require Apelin signals from anterior and posterior to provide directional information. While ubiquitous and vascular reexpression of Apelin partially rescued ISV sprouting, it did not rescue DLAV formation. One possible explanation could be the lack of directional information due to ubiquitous and vascular reexpression, which might be required for the formation of the DLAV but not ISV. Therefore, a possible function of Apelin as a motogen could stimulate tip cell migration during ISV formation independent of possible Apelin gradients. A possible function as a motogen has already been reported during gastrulation for the second Aplnr ligand Apela (*21*). Furthermore, tip cells could be able to self-generate an Apelin gradient (*43*). Because vascular *apln* expression becomes prominent only after the DLAV has formed, this suggests that Apelin derived from ECs may play a role in the remodeling of the DLAV or signal to other cells in close proximity. However, because we observe *aplnrb* expression in our reporter lines mainly in ECs, a possible angiocrine function of vascular-derived Apelin is low but possible. Another possibility is that vascular Apelin is rather a marker of active ECs and has only a minor function on the ECs.

By analyzing the migration patterns of angiogenic sprouts in the zebrafish trunk, we identified four characteristic morphological stages that tip cells go through. Consistent with a previous report (*3*), we detected the formation of long and dominant filopodia toward the dorsal side of the embryo. Apelin signaling is required for the formation of the long filopodia and subsequent elongation of tip cells. Treatment of embryos with latrunculin B to block filopodia formation slows tip cell migration, especially above the horizontal myoseptum (*3*) and both latrunculin B–treated and *apln(rb)* mutant embryos fail to form the DLAV, even when the sprouts reach the dorsal side of the embryo (*3*, *11*), suggesting that Apelin regulates Actin dynamics in tip cells. We found that the long filopodia, which we observed in our study, are similar to specialized protrusions known as dactylopodia in mice, which evolve from filopodia and rely on the activity of the Arp2/3 complex (*34*). Consistently, we observed membrane ruffling and localization of Arp2/3 within the most prominent long filopodium, which then expanded, facilitating the tip cell’s forward movement. This similarity suggests a conserved mechanism across species and underscores the pivotal role of the Arp2/3 complex not only in lamellipodia formation but also in driving cellular migration through structural changes in protrusions. Our observation that tip cells achieve more efficient migration through the employment of long filopodia indicates that these specialized protrusions are highly effective in facilitating cell migration and represents a novel and unique way of cell migration. Dactylopodia formation is counteracted by myosin II activity in tip cells, which promotes smaller filopodia (*34*). The enrichment of myosin at the leading edge of tip cells in *aplnrb* mutant embryos may explain the absence of a dominant filopodium, although smaller filopodia are still present, suggesting that myosin II activity counterbalances the formation of larger, more effective protrusions for migration. Together, our findings imply that Apelin signaling plays a critical role in regulating actin dynamics, triggering the formation of dactylopodia and elongation of tip cells. The interplay of Apelin signaling, myosin II, and the Arp2/3 complex controls the morphological changes required for effective cell migration and provides insights into a sophisticated regulatory mechanism that supports cell migration and vascular morphogenesis.

Our findings position Apelin signaling as an important mediator of sprouting angiogenesis. While loss of Apelin signaling causes severe tip cell defects, it does not cause a complete absence of tip cells or sprouting angiogenesis, as it is the case with the loss of VEGF (*4*, *5*). However, Apelin signaling appears to be able to steer sprouting tip cells and regulate tip cell filopodia formation. If Apelin- and VEGF-dependent filopodia (*1*) are the same type of filopodia requires further research.

In sprouting ECs, VEGF is the main activator of PI3K and MAPK signaling pathways, and loss of VEGF signaling abrogates phosphorylation of ERK (*42*, *44*, *45*). The Aplnr is known to activate PI3K and MAPK signaling pathways in vitro (*37*, *41*). Thus far, an in vivo characterization of the pathways modulated by Apelin signaling in ECs was lacking. We found that Apelin signaling regulates tip cell behaviors by modulating PI3K and ERK activity, two well-known and important drivers of sprouting angiogenesis (*42*, *44*, *46*, *47*). Notably, ERK activity has been shown to be higher in tip cells compared to stalk cells (*10*, *42*, *48*), partially through the asymmetric partitioning of *kdrl* mRNA in tip cells after cell division (*10*). Leveraging advancements in ERK activity sensors (*35*, *36*), we unveiled a critical role for Apelin signaling to maintain high ERK activity in tip cells during sprouting and after tip cell division. However, because inhibition of actin polymerization and cell elongation leads to equal ERK activity in tip and stalk daughter cells, this suggests that Apelin signaling indirectly modulates ERK activity in tip cells through changes in the cytoskeleton and cell shape.

PI3K phosphorylates PIP2 (phosphatidylinositol 4,5-bisphosphate) to generate PIP3, which is enriched at the leading edge of motile cells (*49*). Inhibition of PI3K activity increases EC contractility and prevents formation of the DLAV (*50*), thus phenocopying *apln(rb)* mutant zebrafish. Together, our results suggest that Apelin signaling drives angiogenic sprouting via PI3K and ERK in vivo.

Previously, we have shown that Apelin signaling regulates c-MYC expression in ECs in vitro (*11*). Furthermore, other studies reported that c-MYC expression is downstream of the PI3K-AKT-FOXO1 axis (*51*) and FOXO1 phosphorylation is regulated by Apelin (*52*). This suggests that Apelin signaling may regulate c-MYC expression through the PI3K-AKT-FOXO1 pathway. However, a recent study suggests that high VEGF-Notch signaling inhibit c-MYC expression, which is required during artery formation (*53*). This Notch-mediated regulation may balance tip cell proliferation during sprouting, facilitating tip cells to grow and stretch over long distances.

Asymmetric cell division can result in daughter cells with different fates or different sizes (*54*). Cell size asymmetry of daughter cells has been shown during the division of vascular tip cells (*10*). However, no signaling pathway controlling this process has yet been identified. Here, we identified Apelin signaling to be required for asymmetric daughter cell size after tip cell divisions. In *Caenorhabditis elegans*, it was shown that asymmetric cell division relies on the G protein αi subunit acting as a polarity cue to generate differential pulling forces (*55*). Because the Aplnr couples to the G protein αi subunit, one might speculate that the G protein αi subunit downstream of the Aplnr regulates asymmetric tip cell divisions. Loss of the asymmetric division, which we found to maintain tip cell size, morphology, and ERK activity in agreement with the literature (*10*), likely further exacerbates the tip cell phenotype in *aplnrb* mutants.

The similarity of the signaling cascades activated by Apelin and VEGF signaling pathways supports the notion that these pathways cooperatively regulate angiogenesis. Unlike VEGF signaling, overactivation or loss of Apelin signaling, as shown here or in our previous study (*11*), does not cause ectopic sprouting of the ISVs. This suggests that Apelin signaling mainly adjust rather than induce sprouting. Thus, Apelin signaling may act as an enhancer, fine-tuning ERK and PI3K activity, as well as dactylopodia-like protrusions in tip cells. As a result, tip cells with high Apelin signaling outcompete ECs with lower Apelin signaling (*11*). Loss of Apelin signaling equalizes these differences between tip and stalk cells, resulting in tip-stalk cell shuffling.

In summary, our findings reveal a previously unexplored neurovascular cross-talk, showing that neural progenitor cell–derived Apelin coordinates endothelial sprouting and tip cell functionality.

## MATERIALS AND METHODS

### Zebrafish husbandry

All zebrafish housing and husbandry were performed under standard conditions in accordance with institutional [University of Marburg (UMR)] and national ethical and animal welfare guidelines approved by the ethics committee for animal experiments at the Regierungspräsidium Gießen, Germany, as well as the Federation of European Laboratory Animal Science Associations (FELASA) guidelines (*56*). Embryos were staged by hpf at 28.5°C (*57*). The following lines were used (see Table 1).

**Table 1.** Zebrafish resource table.

Designation	Source or reference	Identifiers
*Tg(fli1a:EGFP)y1*	Lawson and Weinstein (*67*)	ZFIN: y1Tg
*Tg(kdrl:Hsa.HRASmCherry)s896*	Chi *et al.* (*68*)	ZFIN: s896Tg
*Tg(kdrl:NLS-mCherry)is4*	Wang *et al.* (*69*)	ZFIN: is4Tg
*aplnrb mu281*	Helker *et al.* (*11*, *15*)	ZFIN: mu281
*apln mu267*	Helker *et al.* (*15*)	ZFIN: mu267
*Tg(apln:EGFP)bns157*	Helker *et al.* (*11*)	ZFIN: bns157Tg
*Tg(hsp70:apln)mu269*	Helker *et al.* (*11*)	ZFIN: mu269Tg
*Tg(aplnrb:aplnrb-TagRFPsfGFP)bns309*	Helker *et al.* (*11*)	ZFIN: bns309Tg
*Tg(UAS:LIFEACT-EGFP)mu271*	Helker *et al.* (*61*)	ZFIN: mu271Tg
*Tg(fli1a:GAL4FF)ubs4*	Zygmunt *et al.* (*70*)	ZFIN: ubs4Tg
*Tg(elavl3:GAL4-VP16)psi1*	Stevenson *et al.* (*71*)	ZFIN: psi1Tg
*TgBAC(gfap:GAL4FF)s995*	Matsuoka *et al.* (*25*)	ZFIN: s995Tg
*Tg(sox10:GAL4-VP16)km6*	Chung *et al.* (*72*)	ZFIN: km6Tg
*Tg(UAS-E1B:NTR-mCherry)c264Tg*	Davison *et al.* (*73*)	ZFIN: c264Tg
*Tg(5xUAS:RFP-1a)nkuasrfp1a*	Takeuchi *et al.* (*74*)	ZFIN: nkuasrfp1aTg
*Tg(kdrl:myl9a-EGFP) ip5*	Lancino *et al.* (*75*)	ZFIN: ip5Tg
*Tg(fli1a:ERK-KTR-Clover)uq39bh*	Okuda *et al.* (*35*)	ZFIN: uq39bhTg
*TgBAC(nes:EGFP)tud100*	Kaslin *et al.* (*76*)	ZFIN: tud100Tg
*TgBAC(apln:Venus-PEST)mr15*	This manuscript	
*Tg(kdrl:EGFP-CAAX)mr19*	This manuscript	
*Tg(fli1a:PH-AKT-EGFP)mr23*	This manuscript	
*Tg(UAS:apln)mr24*	This manuscript	
*Tg(fli1a:ARPC1B-mVenus)mr25*	This manuscript	
*Tg(−2.1zic2b:GAL4-VP16)mr29*	This manuscript	
*Tg(−2.1zic2b:mCherry)mr30*	This manuscript	

### Generation of transgenic lines

To generate the *apln* BAC construct, we used the published BAC clone from *TgBAC(apln:EGFP)^bns157^* (*11*). All recombineering steps were performed as described by Bussmann and Schulte-Merker (*58*), with the modifications as described by Helker *et al.* (*59*). The following homology arms were used to generate the targeting polymerase chain reaction (PCR) product of the Venus- pest_Kan cassette: apln_HA1_Venus_fw: CCACTACAGTATATCAGCTAGCGACTGGCAGGGAAACGGAGGGGAGAGCAACCATGGTGAGCAAGGGCGAGGAG and apln_HA2_kanR_rev: CACAGCAGAGAAACCACCAGCACAATCACCAGCGTCAAGATCTTCACATTACCATGGAGAAGTTACTATTCCG. The kanamycin cassette was removed with a flippase.

For generation of all other transgenic lines, the *pTol2* or *pminiTol2* vectors were used (*60*). Inserts with overhangs were amplified by PCR with the Takara PrimeSTAR (Takara) polymerase and ligated using In-Fusion cloning (Takara). *Tg(UAS:apln)^mr24^* was generated by replacing the *LIFEACT-EGFP* coding sequence from *pTol2_UAS-LIFEACT-EGFP* (*61*) with the zebrafish *apln* coding sequence from cDNA. *Tg(−2.1 zic2b:mCherry)^mr30^* or *Tg(−2.1 zic2b:GAL4-VP16)^mr29^* were generated by amplifying the sequence 2.1-kb upstream of the *zic2b* coding sequence from zebrafish genomic DNA and replacing the *UAS* promoter of *pTol2_UAS-LIFEACT-EGFP* and subsequently replacing the *LIFEACT-EGFP* coding sequence with the *mCherry* or *GAL4-VP16* coding sequences, respectively. To generate the *Tg(fli1a:PH-AKT-EGFP)^mr23^* and *Tg(fli1a:ARPC1B-mVenus)^mr25^*, either the PH-AKT-EGFP coding sequence was amplified from *pEGFP-N1_PH-AKT-EGFP* (Addgene plasmid no. 51465) or the ARPC1B-mVenus coding sequence was amplified from pEntr3c-Arpc1b-mVenus-P2A-Arpc3-mTurq2 (*62*) and cloned downstream of the *fli1a* promoter. To generate the *Tg(kdrl:EGFP-CAAX)^mr19^*, EGFP-CAAX was amplified and cloned downstream of a 5-kb *kdrl* promoter. All constructs were injected into AB embryos at the one-cell stage (30 pg per embryo) together with Tol2 mRNA (30 pg per embryo) (*63*) to establish to establish stable lines.

### Genotyping

Embryos carrying possible mutations for *apln* or *aplnrb* (*11*) were genotyped by high-resolution melt (HRM) analysis (*64*), and transgenes were identified by PCR: *Tg(UAS:apln)^mr24^*, forward: 5′-TCCGAGCG-GAGACTCTAGAG, reverse: 5′-GTCTCCTCCTCCAGCCAG; *Tg(fli1a:GAL4FF)^ubs4^*, forward: 5′-GCTTTGGGTGCCGTAATGAA, reverse: 5′-AGTAGCGACACTCCCAGTTG; *Tg(elavl3:GAL4-VP16)^psi1^*, forward: 5′-AAACCAAAAGGTCTCCGCTG, reverse: 5′-CCGTCTAAGTGGAGCTCGTC.

### Confocal microscopy

Zebrafish embryos were mounted in 1% low melt or 0.3% normal agarose. E3 and agarose were supplemented with tricaine (19.2 mg/liter). If imaged later after 30 hpf, then embryos were treated with 0.1% (w/v) propylthiouracil from 24 hpf. Fluorescence images were acquired on a Leica Stellaris 8 confocal microscope equipped with HC FLUOTAR L VISIR 25x/0.95 WATER and HC PL APO CS2 40x/1.10 WATER objectives and an Oko-lab incubator set to 28.5°C for time-lapse experiments.

### Filopodia quantification

Filopodia were quantified from maximum intensity projections of confocal stacks acquired from transgenic *Tg(kdrl:EGFP-CAAX)* embryos. Siblings are heterozygous or wild-type for *aplnrb*, and mutants are homozygous (*aplnrb^mu281/mu281^*). Length and the corresponding angle relative to the DA for each filopodium adjacent to the leading edge of the tip cells were measured in Fiji. Filopodia with an angle of 45° to 90° relative to the DA were classified as dorsal, and filopodia with an angle of 0° to 45° were classified as lateral filopodia.

### Quantification of protrusion retractions

Protrusions formed or retracted by tip cells (excluding filopodia) were observed between 23 and 30 hpf for wild-type or drug-treated embryos or due to delayed protrusion formation between 30 and 39 hpf for *aplnrb^mu281/mu281^* embryos. Same control embryos were used in Fig. 5D and fig. S12D.

### Line graph intensity measurements

Line graphs of fluorescence intensity [for *Tg(kdrl:myl9a-EGFP)^ip5Tg^* or *Tg(fli1a:PH-AKT-EGFP)^mr23^*] were generated by measuring the fluorescence intensity (in confocal maximum intensity projections) from the leading edge of the tip cell toward the center of the cell in Fiji. The values for each distance were then normalized to the average intensity across the whole line.

### Cell tracking

The migration of tip cells was measured by using the spots function in Imaris 9.7.2. The linear distance in dorsal direction between the leading edge of the tip cell (excluding filopodial protrusions) and the aorta was measured (dorsal distance).

### Mosaic tip cell labeling

To label individual tip cells, a plasmid was generated that expresses nuclear BFP in one and GFP-CAAX in the other direction driven by a bidirectional *fli1a* promoter (*BFP-nls:fli1a:EGFP-CAAX*) (*65*). The construct was injected in the offspring of an *aplnrb^wt/mu281^ Tg(kdrl:Hsa.HRAS-mCherry)^s896^* incross at one-cell stage. Embryos were imaged the next day at 25 and 30 hpf.

Cell volumes were measured in Imaris 9.7.2 by generating a surface of tip cells positive for the GFP-CAAX signal. Cell volumes were normalized to sibling cell size at 25 hpf.

### *Tg(hsp70:apln)* overexpression experiments

For the temporal ubiquitous overexpression of Apelin via the *Tg(hsp70:apln)^mu269^* transgene, *apln^wt/mu267^ Tg(hsp70:apln)^mu269^* fish were outcrossed to *apln^wt/mu267^* fish that do not carry the heat shock transgene. The offspring was then subjected to a single heat shock at 37°C for 60 min at 24 hpf and imaged under the confocal microscope at 32 hpf.

### Tissue-specific *apln* rescue experiments

For the rescue experiment, *apln^wt/mu267^ Tg(UAS:apln)^mr24^* fish were crossed to either *apln^wt/mu267^*; *Tg(fli1a:GAL4FF)^ubs4^*; *Tg(UAS:LIFEACT-EGFP)^mu271^/Tg(fli1a:EGFP)^y1^* (vascular overexpression) or *apln^wt/mu267^*; *Tg(zic2b:GAL4-VP16)^mr29^*; *Tg(fli1a:EGFP)^y1^* (neural overexpression) or *apln^wt/mu267^*; *Tg(elavl3:GAL4-VP16)^psi1^*; *Tg(fli1a:EGFP)^y1^* (neuronal overexpression). Offspring was imaged at 32 hpf and genotyped (see the “Genotyping” section). *apln^mu267/mu267^* mutant embryos negative for *Tg(UAS:apln)^mr24^* were used as a control.

### Quantification of ERK activity in vivo

Nuclear signal of the ERK activity reporter [*Tg(fli1a:ERK-KTR-Clover)*] was measured in Imaris 9.7.2 as described in (*35*). Two to five tip cells of adjacent ISVs above the yolk extension were analyzed per embryo. Intensities were normalized to the average nuclear ERK intensity of the aorta section adjacent to the analyzed ISVs. To generate the color-coded nuclei, the Clover channel was masked with the generated surface, transferred to Fiji, and converted to an 8-bit image and the Lookup Table (LUT) was set to “16 colors.”

To measure ERK activity after cell division, confocal time-lapse videos of *Tg(fli1a:ERK-KTR-Clover)* of *aplnrb* mutant embryos or wild-type embryos were analyzed between 25 and 27 hpf. Nuclear intensity of the ERK reporter was measured (as described above) 35 to 45 min after tip cell division.

### crRNA design

To generate P0 knockouts of the *pik3cg* and *pik3cb* genes, three synthetic CRISPR RNAs (crRNAs) targeting different exons were annealed to trans-activating RNA (trRNA) and coinjected into one-cell stage embryos with Cas9 protein as described previously (*40*). Cas9 protein without crRNA-trRNA was used as a control. Before the experiments, functionality of the crRNAs was tested by HRM analysis.

Synthetic RNA and Cas9 protein were purchased from IDT. Pre-designed crRNAs were chosen if available; otherwise, custom crRNAs were designed with the IDT web tool. The crRNAs were selected based on highest on/off-target scores. The following crRNAs were used (see Table 2).

**Table 2.** crRNA sequences.

crRNA name	Location	Target sequence [PAM]	HRM forward primer	HRM reverse primer
pik3cg crRNA 1	Exon 2	GGATCCCAACCTTTATTCGA [TGG]	AGAAAGCTACTGACCCCTCG	TGAGCAAGTATTCAGGCATTGG
pik3cg crRNA 2	Exon 7	ACTTATTATGGAGTCTATCT [GGG]	GCAGTCATCATCCTAATTGTCCA	CCAGTGGAAATGCAGCCG
pik3cg crRNA 3	Exon 9	TGTGACGATCACCAATGCCA [AGG]	TCGTGTGGAGGGTATTGTGT	AGAGCTTAAACAGAACCCGGA
pik3cb crRNA 1	Exon 5	TCCAAGATTGGAGTTCTCAT [TGG]	TAATTGTGGACGTGCAGAGC	GCGTGTGATTATGTACATATGCG
pik3cb crRNA 2	Exon 7	TGGATCATGCTCTCAAGAAA [TGG]	CCAGCCTGAAAGTATCGTTGA	AATGTCTTCCTCTGGCCCAT
pik3cb crRNA 3	Exon 22	ACCAATGCCCAAGACATAGG [TGG]	ACAGGGATGCTTTGGACAAA	CCCTGTACTGCGGACCATTA

### Inhibitor treatments

Embryos were treated at the indicated time points with the indicated concentrations. The equivalent amount of dimethyl sulfoxide (DMSO) was used for control treatments. The following inhibitors were used: CK666 from Sigma-Aldrich (catalog no. SML0006) (10 mM stock; 100 μM working concentration), latrunculin B from Abcam (catalog no. ab144291) (50 μM stock; 125 or 375 nM working concentration), LY294002 from Cell Signaling (catalog no. 9901) (10 mM stock; 15 or 25 μM working concentration), and AS-605240 from Selleckchem (catalog no. S1410) (5 mM stock; 300 nM working concentration).

For “low-dose treatments,” a dose of the respective inhibitor that did not cause an obvious phenotype in wild-type embryos was determined. Next, *aplnrb^wt/mu281^ Tg(fli1a:EGFP)^y1^* fish were outcrossed to wild-type fish and the offspring treated from 22 to 42 hpf. Embryos were analyzed under a fluorescence microscope and grouped into different categories (see Quantification of phenotypes) and genotyped.

### Quantification of phenotypes

For every embryo, somites 5 to 15 were analyzed: normal (no phenotype): 10 fully developed ISVs and connected DLAV; mild: ISVs fully outgrown but 1 to 5 gaps in the DLAV; medium: ISVs fully outgrown but more than 5 gaps in the DLAV; and severe: 1 to 6 ISVs shortened/truncated.

### Whole-mount immunohistochemistry

Embryos were fixed in 4% paraformaldehyde at 4°C overnight, washed with phosphate-buffered saline (PBS) with 0.1% Tween (PBSTw), and stored in methanol. For staining, embryos were rehydrated with PBSTw, permeabilized with water then PBSTw with 1% Triton, and then treated with proteinase K (10 ng/μl; 5 min). After antigen retrieval by treating with citrate buffer (0.1 M trisodium citrate dihydrate and 0.05% Tween 20; pH 6.0) at 96°C for 20 min, embryos were incubated in blocking solution (0.1% PBSTw, 0,5% Triton X-100, 10% DMSO, 1% goat serum, and 5% bovine serum albumin) for 2 hours at room temperature. Then, embryos were incubated with primary antibodies chicken polyclonal anti-GFP 1:500 (Abcam, ab13970) and rabbit polyclonal anti–glial fibrillary acidic protein (GFAP) 1:500 (Dako GA52461-2) in blocking solution at 4°C for 2 days. After washing with PBSTw, embryos were incubated with secondary antibodies (mouse anti-chicken AF 488 1:500 and mouse anti-rabbit AF 568 1:500) at 4°C in blocking solution overnight. Embryos were then washed with PBSTw and stained with DAPI (4′,6-diamidino-2-phenylindole).

### Whole-mount in situ hybridization

In situ hybridizations were performed as described previously (*61*, *66*). The following probes were synthesized: *apln* (*15*) and *aplnrb* (*15*).

### Statistics and reproducibility

Statistical analysis was performed in Prism 9 (GraphPad). Respective tests and *n* numbers are indicated in the figure legends. For normally distributed data, we used two-tailed unpaired Student’s *t* test to compare between two means and one-way analysis of variance (ANOVA) with Tukey’s multiple comparison test for comparison of multiple conditions. For non-normally distributed data, Mann-Whitney test was used to compare between two means and one-way ANOVA with Kruskal-Wallis test was used for comparison of multiple conditions. For grouped data, two-way ANOVA with Tukey’s multiple comparison test or ANOVA with Bonferroni correction (when comparing selected values of multiple conditions, e.g., two conditions over time; Fig. 7F) was used. Values are shown as mean ± SD. *P* values of <0.05 were deemed as significant.
