# Supplementary material for: Neural progenitor–derived Apelin controls tip cell behavior and vascular patterning
Source: Sci Adv. 2024 Jul 5;10(27):eadk1174. doi: 10.1126/sciadv.adk1174 (PMC11225789; doi:10.1126/sciadv.adk1174)
Supplement: Supplementary file 1 — Figs. S1 to S14 Legends for movies S1 to S8 [file sciadv.adk1174_sm.pdf]

Science Advances
AAAS

Supplementary Materials for

Neural progenitor–derived Apelin controls tip cell behavior and vascular patterning

Julian Malchow *et al.*

Corresponding author: Christian S. M. Helker, christian.helker@biologie.uni-marburg.de

Sci. Adv. **10**, eadk1174 (2024)
DOI: 10.1126/sciadv.adk1174

The PDF file includes:

Figs. S1 to S14
Legends for movies S1 to S8

Other Supplementary Material for this manuscript includes the following:

Movies S1 to S8

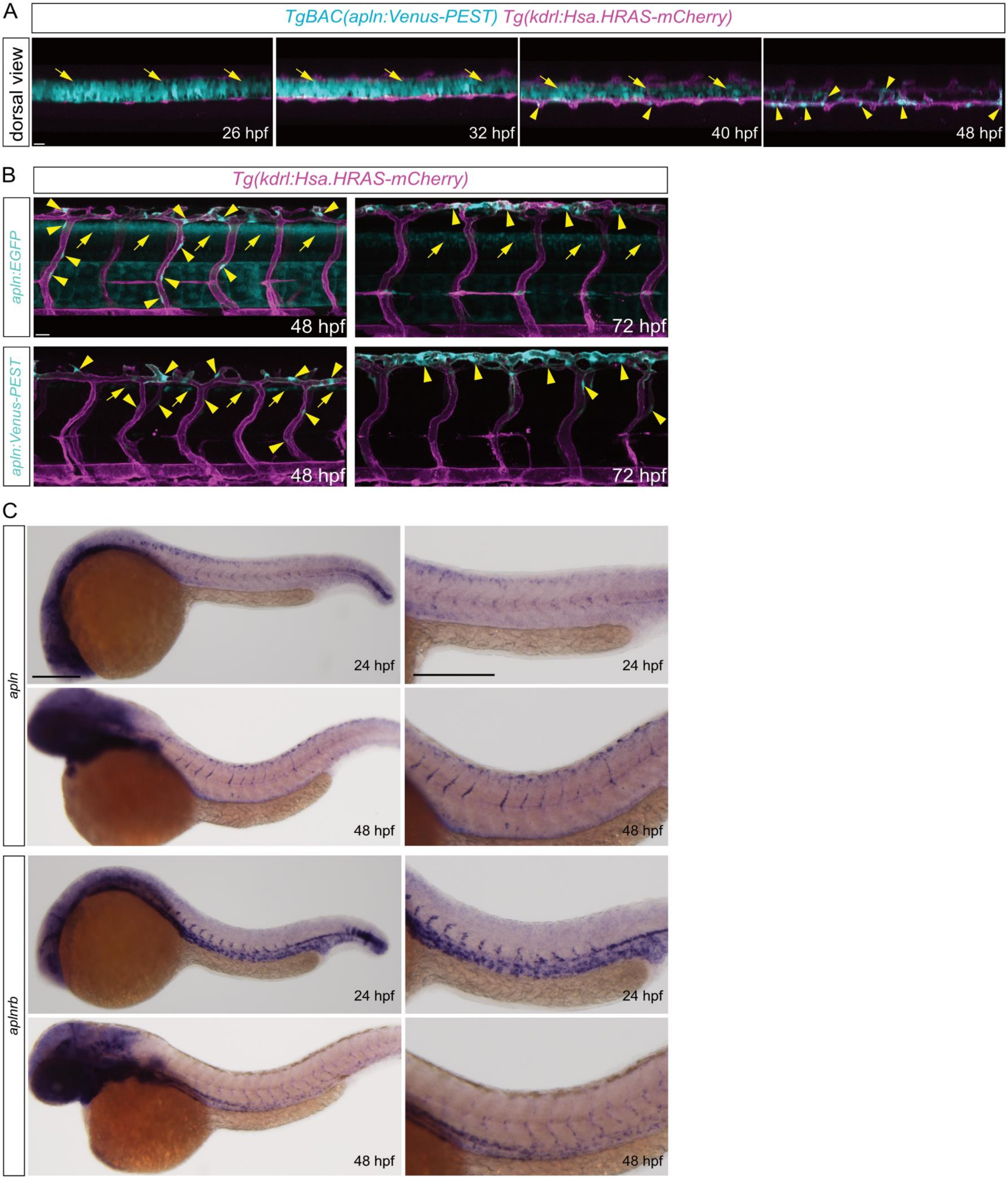
A
TgBAC(apln:Venus-PEST) Tg(kdrl:Hsa.HRAS-mCherry)
dorsal view
26 hpf
32 hpf
40 hpf
48 hpf
B
Tg(kdrl:Hsa.HRAS-mCherry)
apln:EGFP
48 hpf
72 hpf
apln:Venus-PEST
48 hpf
72 hpf
C
apln
24 hpf
24 hpf
48 hpf
48 hpf
aplnrb
24 hpf
24 hpf
48 hpf
48 hpf

fig. S1. *apln* and *aplnrb* expression during ISV sprouting. (**A**) Still images taken from a time-lapse video of sprouting ISVs of *TgBAC(apln:Venus-PEST)*; *Tg(kdrl:HsHRAS-mCherry)* embryos. Dorsal view of the time-lapse of Fig. 1 A. *apln:Venus-PEST* expression in the neural tube (arrows) and ECs (arrowheads). (**B**) Comparison of the *TgBAC(apln:EGFP)* and the *TgBAC(apln:Venus-PEST)* reporter at 48 and 72 hpf. Arrows point towards signal in the neural tube, arrowheads point towards signal in the vasculature. (**C**) Expression of *apln* and *aplnrb* by whole-mount *in situ* hybridization at 24 or 48 hpf. Scale Bars: 200 µm (C), 20 µm (A), (B).

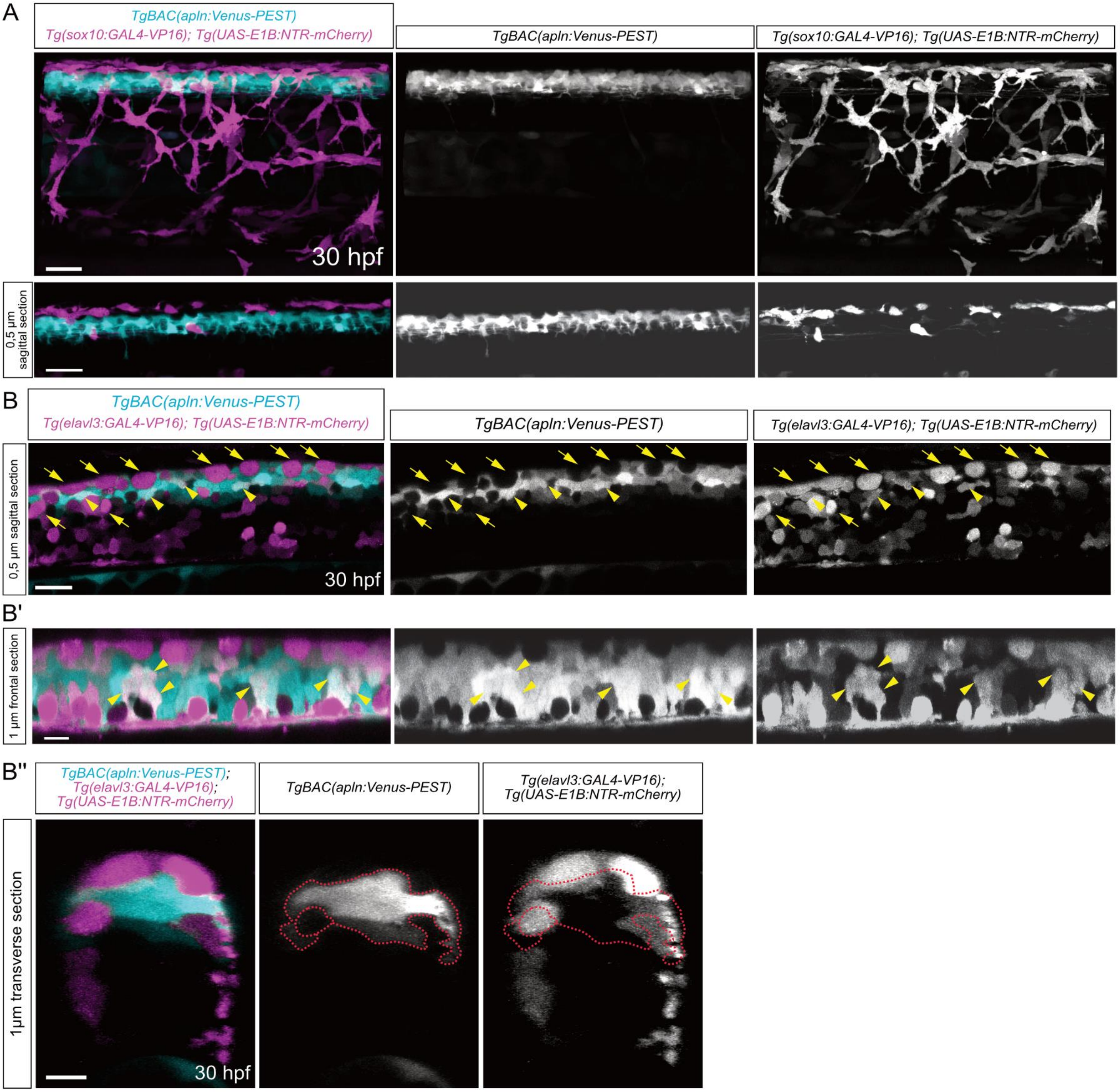

fig. S2: *apln:*Venus-PEST expression in a few neurons but not neural crest cells (A) Confocal projections of *TgBAC(apln:Venus-PEST)*; *Tg(sox10:GAL4-VP16); Tg(UAS-E1B:NTR-*

mCherry) transgenic embryos. (B – B'') Confocal images of *TgBAC(apln:Venus-PEST)*; *Tg(elavl3:GAL4-VP16)*; *Tg(UAS-E1B:NTR-mCherry)* embryos at 30 hpf. Arrows point towards mCherry positive, Venus negative neurons. Arrowheads point towards double positive cells. (**B**) sagittal section. (**B'**) frontal section. (**B''**) transverse section. Red dashed line outlines *apln*:Venus-PEST expression. Scale Bars: 30 µm (A), 20 µm (B), 10 µm (B'), (B'').

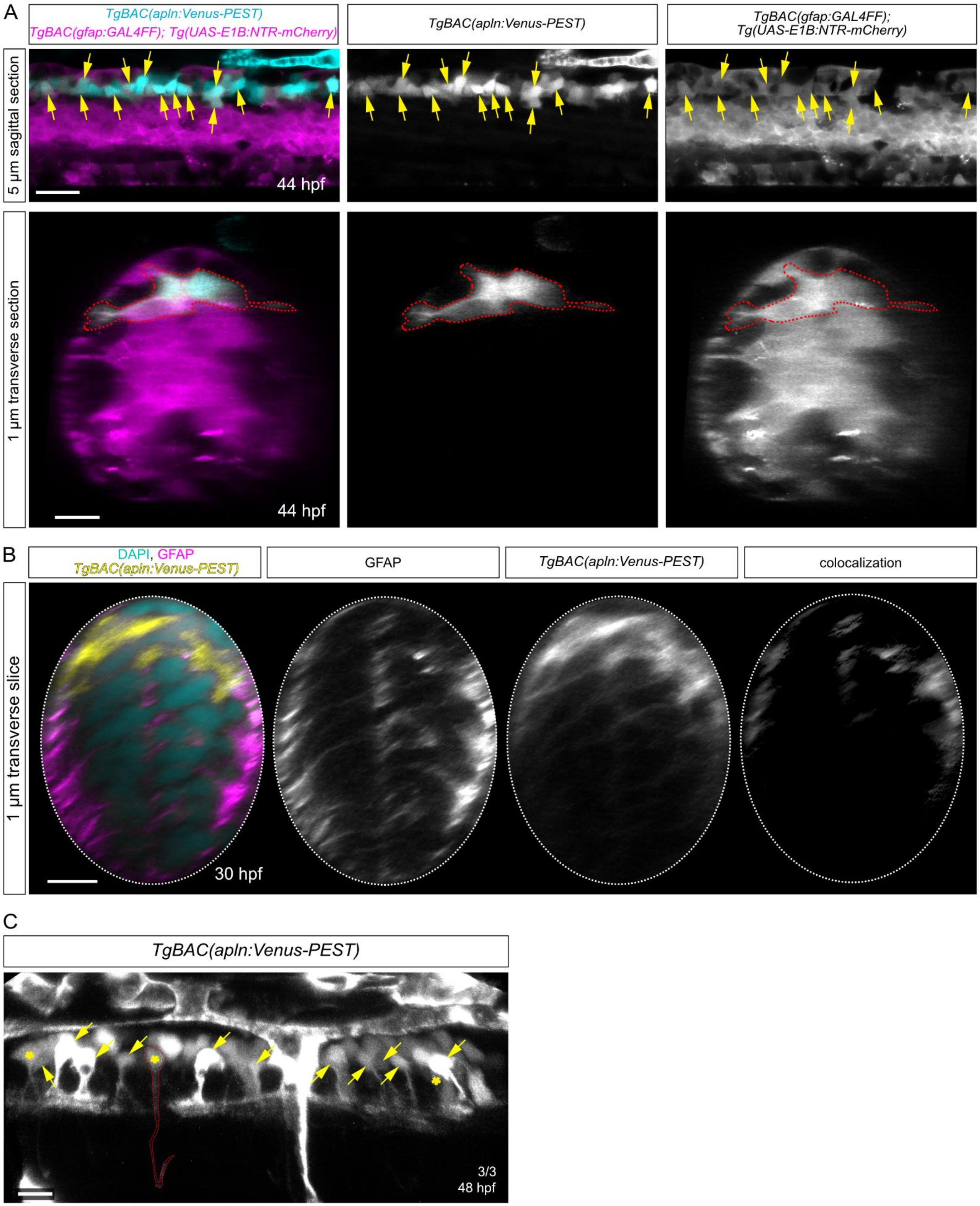
A
TgBAC(apln:Venus-PEST)
TgBAC(gfap:GAL4FF); Tg(UAS-E1B:NTR-mCherry)
TgBAC(apln:Venus-PEST)
TgBAC(gfap:GAL4FF);
Tg(UAS-E1B:NTR-mCherry)
5 µm sagittal section
44 hpf
1 µm transverse section
44 hpf
B
DAPI, GFAP
TgBAC(apln:Venus-PEST)
GFAP
TgBAC(apln:Venus-PEST)
colocalization
1 µm transverse slice
30 hpf
C
TgBAC(apln:Venus-PEST)
3/3
48 hpf

fig. S3: *apln* is expressed in *gfap*-expressing neural cells. (**A**) High-resolution sagittal and transverse confocal sections of the neural tube of *TgBAC(apln:Venus-PEST); TgBAC(gfap:GAL4FF); Tg(UAS-E1B:NTR-mCherry)* embryos. Sagittal section: arrows mark *apln:*Venus-PEST and *gfap:*GAL4FF; *UAS-E1B:*NTR-mCherry double positive cells. Transverse section: red dashed line outlines *apln*:Venus-PEST expression. (**B**) Transverse section of the neural tube. Whole-mount immunostaining of embryos expressing *TgBAC(apln:Venus-PEST)* at 30 hpf stained with anti Gfap, anti GFP (Venus) and DAPI. Colocalization channel of Gfap and Venus shown on the right. White dashed line outlines the neural tube. (**C**) Confocal projections of a *TgBAC(apln:Venus-PEST)* embryo at 48 hpf (dorsolateral view). *apln*:Venus-PEST-expressing cells exhibit a radial glia-like morphology (arrows). Asterisks point to neurons that extend their axons ventrally (an exemplary neuron is outlined by the red dashed line). N = 3 embryos. Scale Bars: 20 µm (A),10µm (A’), (B).

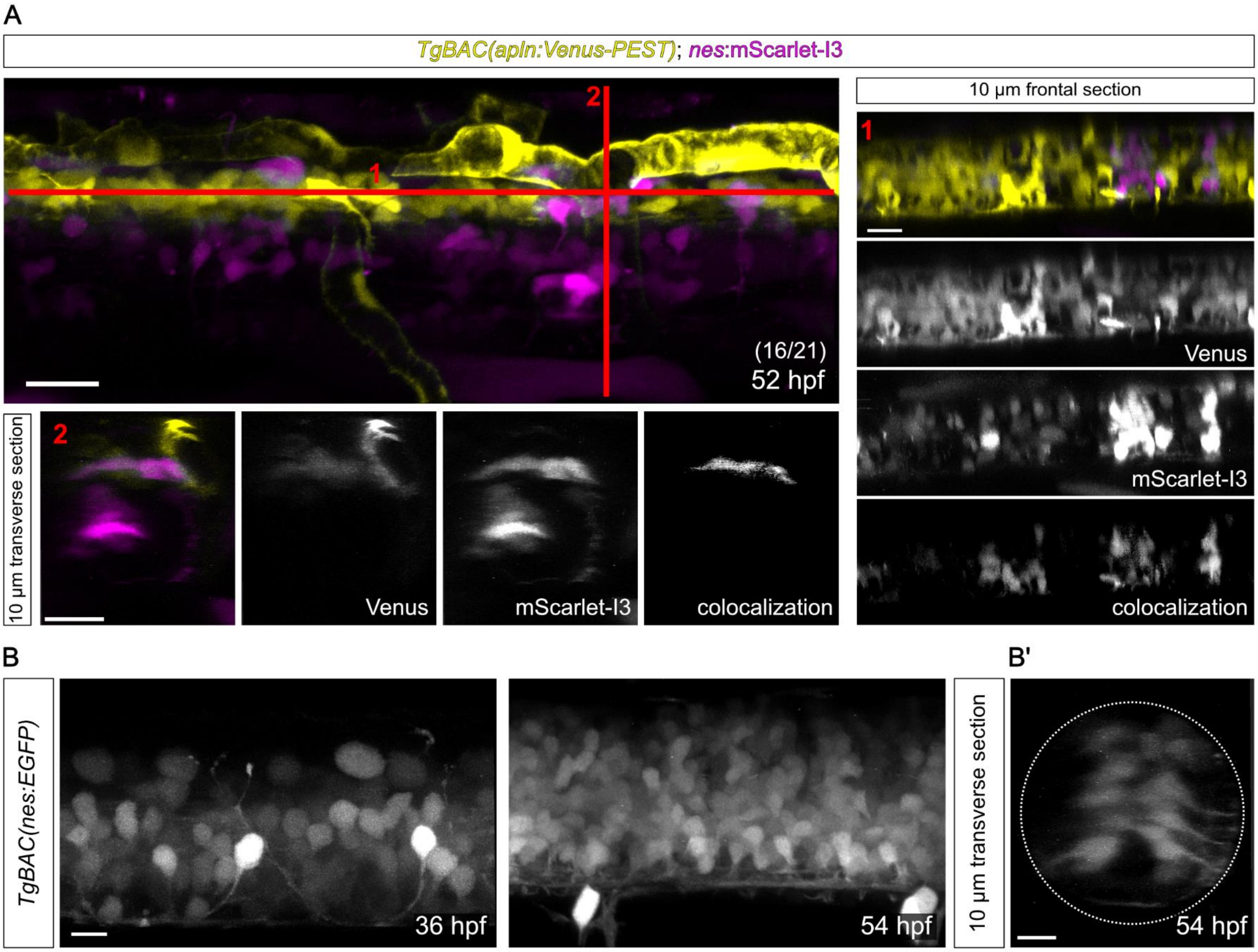

fig. S4: Coexpresion of *apln*:Venus-PEST with *nestin*. (**A**) Confocal images of a *TgBAC(apln:Venus-PEST)* embryo at 52 hpf injected with a *nes:mScarlet-I3* plasmid. 1 frontal slice, 2 transverse slice. N = 21 embryos. (**B**) Confocal projections of *TgBAC(nes:EGFP)* (**B'**) Transverse section. Scale Bars: 20 µm (A), 10 µm (B), (B').

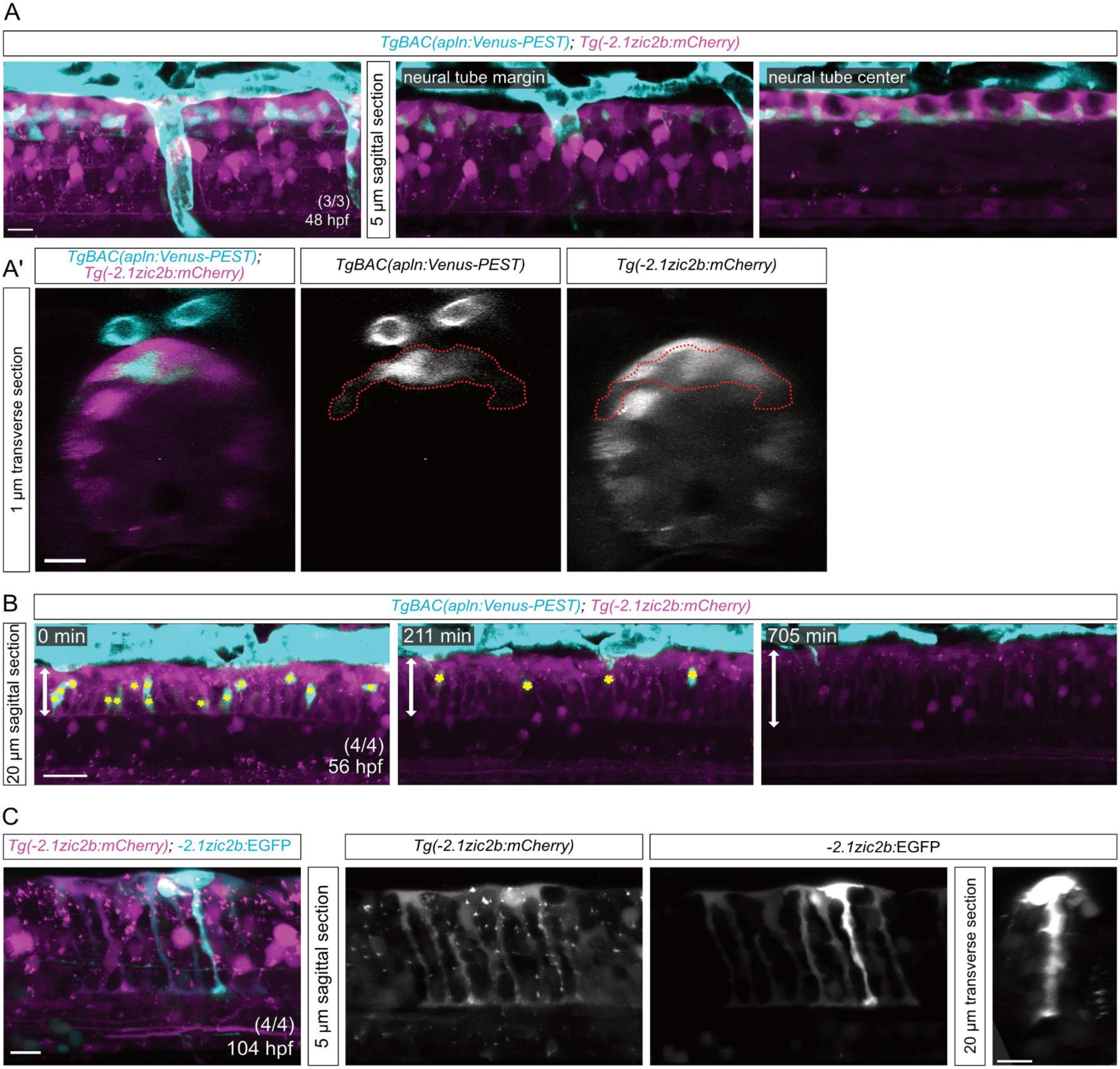

fig. S5: *zic2b*.mCherry expression in roof plate cells, dorsal progenitor cells and interneurons. (**A**) Confocal images of a *TgBAC(apln:Venus-PEST)*; *Tg(-2.1zic2b:mCherry)* embryo at 48 hpf. Sagittal sections showing *zic2b*-expressing interneurons in the outer neural

tube and *zic2b*-expressing in roof plate cells in the central neural tube. (**A'**) Transverse section. *apln*:Venus-PEST expressing cells outlined by red dashed line. N = 3 embryos. (**B**) Sagittal sections from a time-lapse movie of *TgBAC(apln:Venus-PEST)*; *Tg(-2.1zic2b:mCherry)* embryos starting at 56 hpf. Yellow asterisks point to *apln*:Venus-PEST; *zic2b*:mCherry expressing cells; white arrow depicts ventral extension of the roof plate cells. (**C**) Confocal images of a *Tg(-2.1zic2b:mCherry)* embryo at 104 hpf injected with *-2.1zic2b:EGFP*. Scale Bars: 20 µm (B), 10 µm (A), (A'), (C).

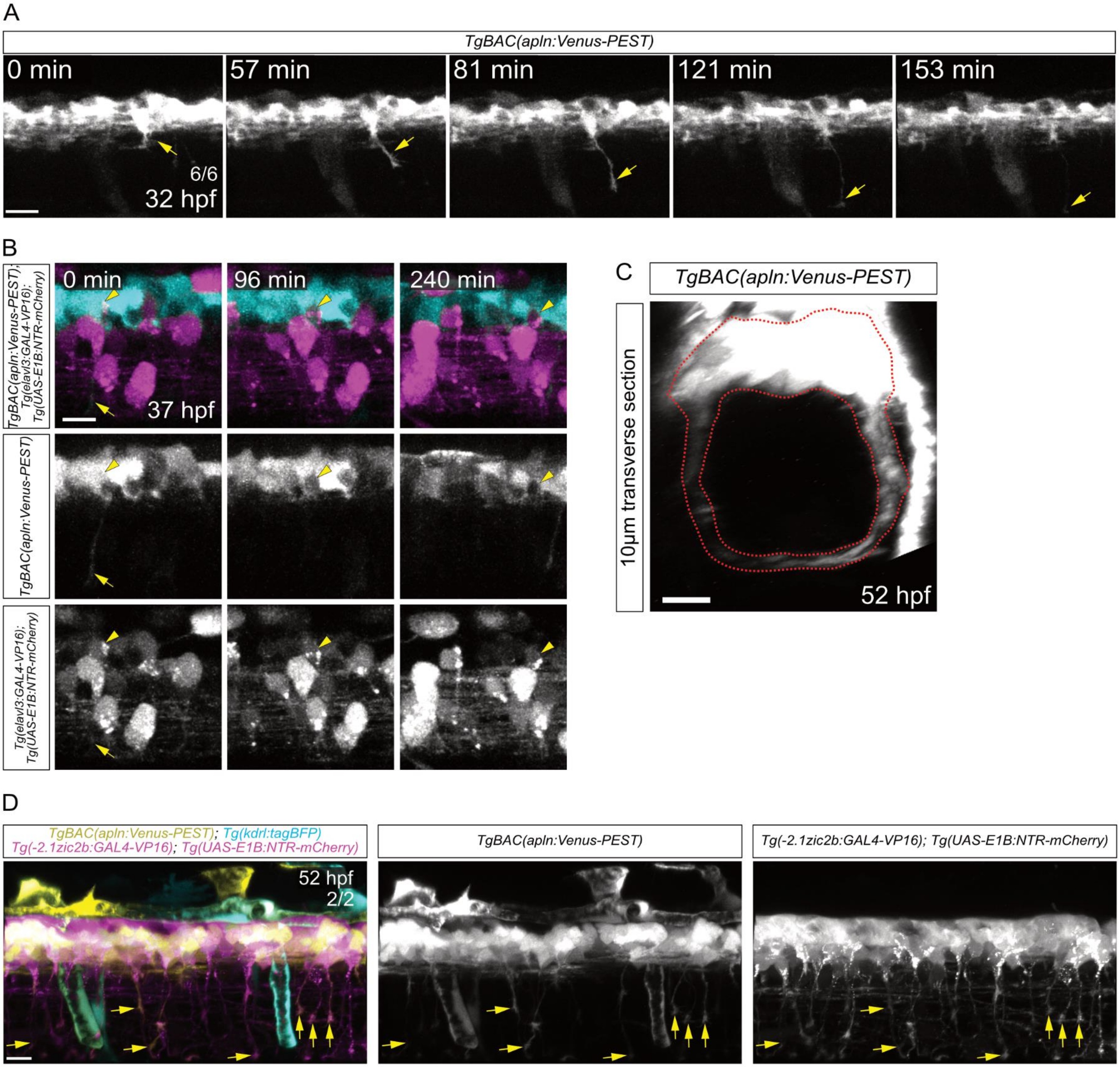

fig. S6: *apln:*Venus-PEST-expressing cells give rise to interneurons. (**A**) Still images from a confocal time-lapse movie of a *TgBAC(apln:Venus-PEST)* embryo. Arrow point to a axon arising from an *apln*-expressing cell. N = 6 embryos. (**B**) Still images from a confocal time-lapse

movie of *TgBAC(apln:Venus-PEST)*; *Tg(elavl3:GAL4-VP16)*; *Tg(UAS-E1B:NTR-mCherry)* embryo starting at 37 hpf. Arrow points towards axon of *apln:*Venus-PEST and *elavl3:*GAL4-VP16; *UAS-E1B:*NTR-mCherry-expressing cell, which turns off the expression of *apln:*Venus-PEST; arrowhead labels nucleus of the same cell. (**C**) Transverse section of a *TgBAC(apln:Venus-PEST)* embryo at 52 hpf. Red dashed line outlines *apln*:Venus-PEST signal. (**D**) Representative confocal projections taken from *TgBAC(apln:Venus-PEST)*; *Tg(kdrl:tagBFP)*; *TgBAC(zic2b:GAL4-VP16)*; *Tg(UAS-E1B:NTR-mCherry)* embryos at 52 hpf. *zic2b*:GAL4-VP16; *UAS-E1B*:NTR-mCherry expression (yellow arrwos) in interneurons with ventrally extending axons. N = 2 embryos. Scale Bars: 20 µm (A),10 µm (B), (C), (D).

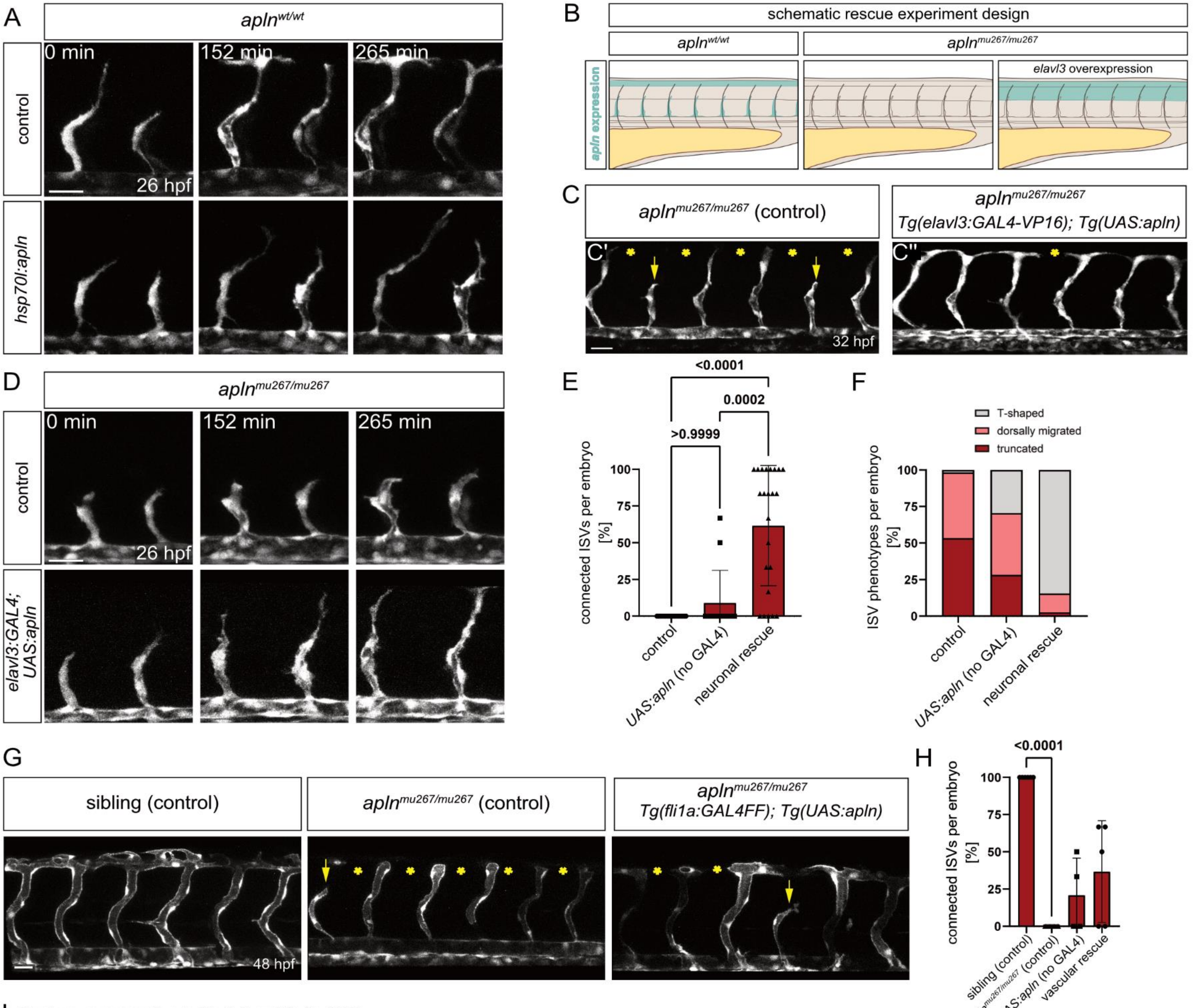

P-values corresponding to Fig. 3 D and G; fig. S7 F

T-shape	Adjusted P Value	T-shape	Adjusted P Value	T-shape	Adjusted P Value
sibling vs. $apln^{mu267/mu267}$	<0,0001	control vs. *zic2b:GAL4; UAS:apln*	<0,0001	control vs. *UAS:apln* (no GAL4)	0,0003
sibling vs. sibling; *hsp70l:apln*	<0,0001	control vs. *fli1a:GAL4; UAS:apln*	<0,0001	control vs. *elavl3:GAL4; UAS:apln*	<0,0001
sibling vs. $apln^{mu267/mu267}$; *hsp70l:apln*	<0,0001	*zic2b:GAL4; UAS:apln* vs. *fli1a:GAL4; UAS:apln*	<0,0001	*UAS:apln* (no GAL4) vs. *elavl3:GAL4; UAS:apln*	<0,0001
$apln^{mu267/mu267}$ vs. sibling; *hsp70l:apln*	<0,0001				
$apln^{mu267/mu267}$ vs. $apln^{mu267/mu267}$; *hsp70l:apln*	0,0408				
sibling; *hsp70l:apln* vs. $apln^{mu267/mu267}$; *hsp70l:apln*	0,6286				
dorsally migrated		dorsally migrated		dorsally migrated	
sibling vs. $apln^{mu267/mu267}$	0,0028	control vs. *zic2b:GAL4; UAS:apln*	0,4021	control vs. *UAS:apln* (no GAL4)	0,922
sibling vs. sibling; *hsp70l:apln*	<0,0001	control vs. *fli1a:GAL4; UAS:apln*	0,0006	control vs. *elavl3:GAL4; UAS:apln*	<0,0001
sibling vs. $apln^{mu267/mu267}$; *hsp70l:apln*	<0,0001	*zic2b:GAL4; UAS:apln* vs. *fli1a:GAL4; UAS:apln*	0,0001	*UAS:apln* (no GAL4) vs. *elavl3:GAL4; UAS:apln*	0,0002
$apln^{mu267/mu267}$ vs. sibling; *hsp70l:apln*	0,0084				
$apln^{mu267/mu267}$ vs. $apln^{mu267/mu267}$; *hsp70l:apln*	0,0673				
sibling; *hsp70l:apln* vs. $apln^{mu267/mu267}$; *hsp70l:apln*	0,9949				
truncated		truncated		truncated	
sibling vs. $apln^{mu267/mu267}$	<0,0001	control vs. *zic2b:GAL4; UAS:apln*	<0,0001	control vs. *UAS:apln* (no GAL4)	0,0012
sibling vs. sibling; *hsp70l:apln*	0,0328	control vs. *fli1a:GAL4; UAS:apln*	<0,0001	control vs. *elavl3:GAL4; UAS:apln*	<0,0001
sibling vs. $apln^{mu267/mu267}$; *hsp70l:apln*	0,0025	*zic2b:GAL4; UAS:apln* vs. *fli1a:GAL4; UAS:apln*	0,1991	*UAS:apln* (no GAL4) vs. *elavl3:GAL4; UAS:apln*	0,0016
$apln^{mu267/mu267}$ vs. sibling; *hsp70l:apln*	<0,0001				
$apln^{mu267/mu267}$ vs. $apln^{mu267/mu267}$; *hsp70l:apln*	<0,0001				
sibling; *hsp70l:apln* vs. $apln^{mu267/mu267}$; *hsp70l:apln*	0,4741				

fig. S7: Neural tube derived Apelin is sufficient to rescue formation of the DLAV. (A, C, D, G) Confocal projections of the zebrafish trunk vasculature labeled by *Tg(fli1a:EGFP)* at the indicated stages. (**A**) Still images from a time-lapse movie of *Tg(hsp70l:apln*) embryos. (**B**) Schematic model of the neural tube *apln* expression rescue experiment. (**C**) Neural tube expression of *apln*, by using *Tg(elavl3:GAL4-VP16)*; *Tg(UAS:apln)* embryos, rescues the dorsal migration of tip cells and DLAV formation in *apln* mutants. Asterisks point to missing DLAV segments; arrows point to truncated ISVs. (**D**) Still images from a time-lapse movie of a *elavl3:*GAL4-VP16; *UAS:*apln expressing *apln*$^{mu267/mu267}$ embryo. (**E**) Percentage of ISVs that formed at least one DLAV connection. (**F**) Percentage of ISVs displaying one of the three indicated vessel phenotypes. (E, F) Control N = 29 embryos, *Tg(UAS:apln)* positive (no GAL4 driver) N = 13 embryos, neuronal overexpression N = 23 embryos; 6 ISVs per embryo from ≥ 4 experiments; (E) P-values calculated by ANOVA with Kruskal–Wallis test. (F) P-values calculated by ANOVA with Tukey's test. (**G**) Vascular expression of *apln*, by using *Tg(fli1a:GAL4FF)*; *Tg(UAS:apln)* embryos, only partially rescues the dorsal migration of tip cells but not DLAV formation in *apln* mutants at 48 hpf. Asterisks point to missing DLAV segments; arrows point to truncated ISVs. (**H**) Percentage of ISVs that formed at least one DLAV connection. P-values calculated by ANOVA with Kruskal–Wallis test. Sibling control N = 6 embryos, *apln*$^{mu267/mu267}$ control N = 11 embryos, *Tg(UAS:apln)* positive (no GAL4 driver) N = 4 embryos; vascular *apln* overexpression N = 5 embryos. (**I**) P-values of the experiments in Fig. 3 D (left), Fig. 3 G (middle) and fig. S7 F (right). Scale Bars: 30 µm.

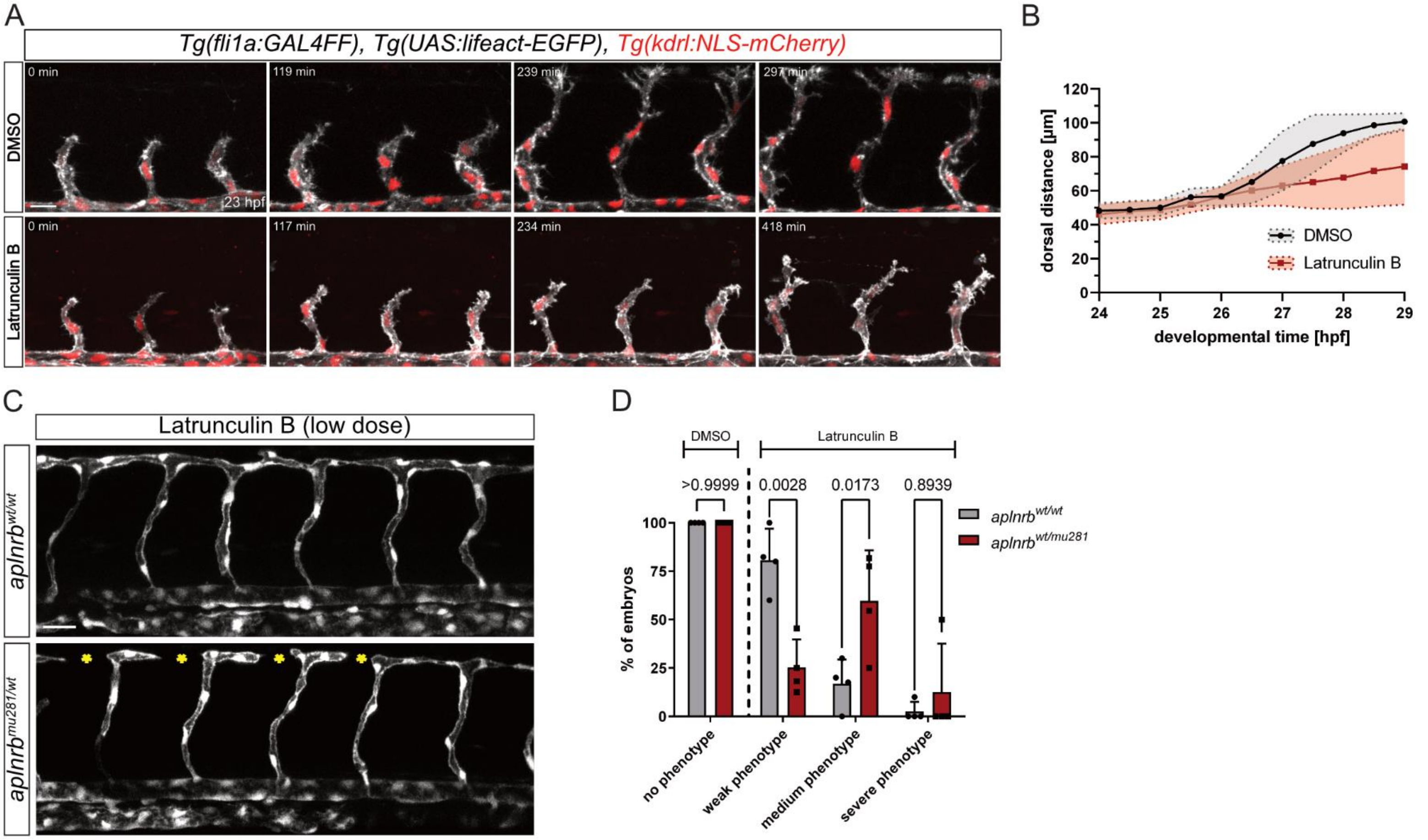

fig. S8: Tip cell elongation requires filopodia and actin polymerization. (**A**) Still images taken from time-lapse videos of *Tg(fli1a:GAL4FF)*; *Tg(UAS:LIFEACT-EGFP)*; *Tg(kdrl:NLS-mCherry)* embryos. Embryos were treated with 375 nM Latrunculin B or DMSO as a control 30 min prior the first frame. (**B**) Dorsal displacement of tip cells from 24 hpf – 29 hpf in Latrunculin B and DMSO treated embryos. Outline is the standard deviation. DMSO: n = 10 ISVs of N = 4 embryos. Latrunculin B: n = 12 ISVs of N = 2 embryos. (**C**) Confocal images of *Tg(fli1a:EGFP)* expressing *aplnrb*$^{wt/wt}$ or *aplnrb*$^{wt/mu281}$ embryos treated with a low dose (125 nM) of Latrunculin B. Asterisks point to missing DLAV segments. (**D**) Analysis resulted in different phenotypic categories according to the extent of ISV development. *aplnrb*$^{wt/wt}$ n = 38 embryos from N = 4 experiments. *aplnrb*$^{wt/wt}$ n = 38 embryos from N = 4 experiments. *aplnrb*$^{wt/mu281}$ n = 38 embryos from N = 4 experiments. P-values calculated by ANOVA with Tukey's test. Scale Bars: 20 µm.

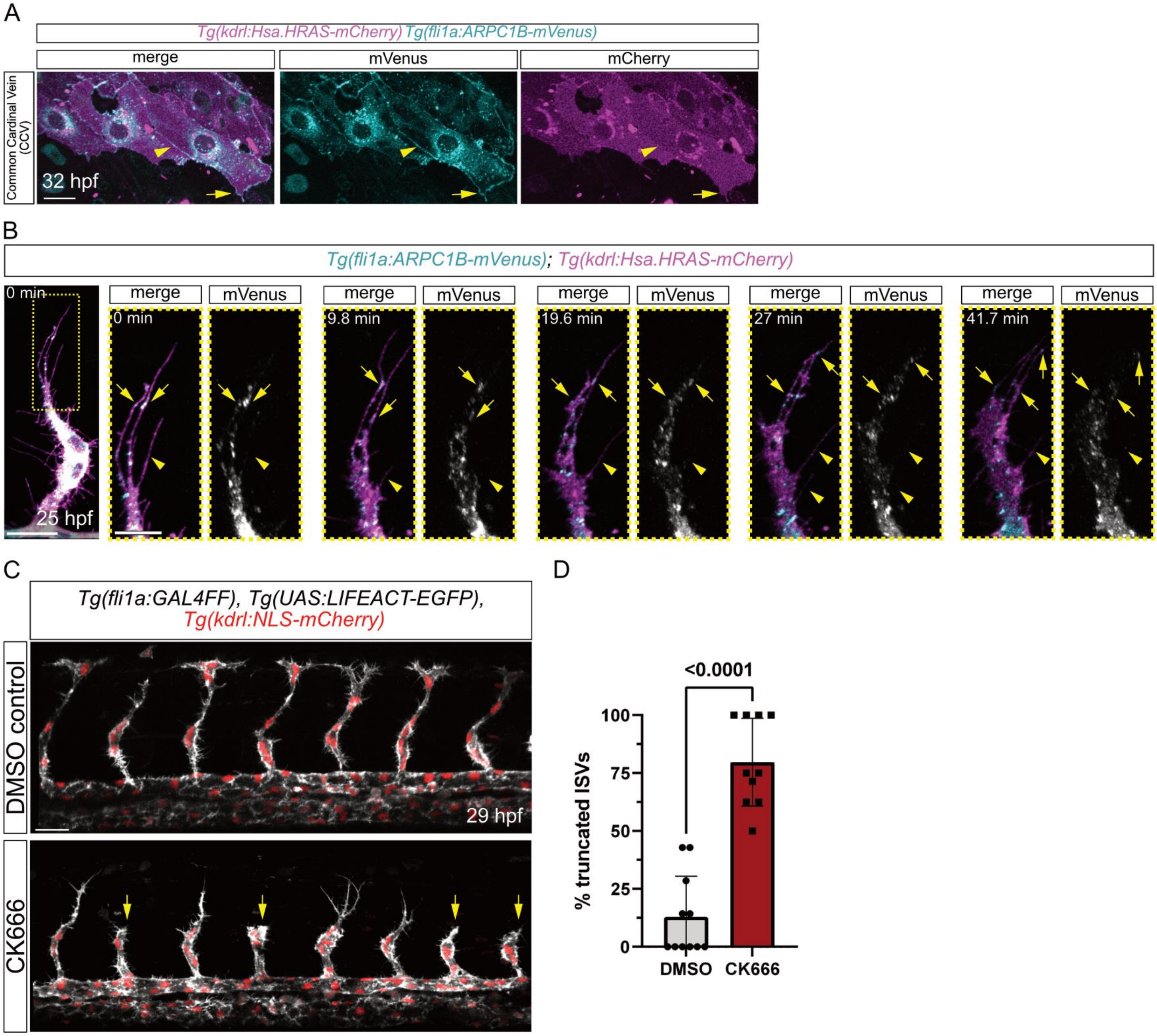

fig. S9: The Arp2/3 complex localizes to long filopodia and is required for tip cell elongation.

(**A**) ARPC1B-mVenus localization at cell-cell junctions (arrowheads) and lamellipodia (arrows) in endothelial cells of the common cardinal vein (CCV). (**B**) Still images taken from a time-lapse video of a *Tg(fli1a:ARPC1B-mVenus)*; *Tg(kdrl:Hsa.HRAS-mCherry*) expressing embryo. Arrows point towards ARPC1B-mVenus localization in long filopodia. Arrowheads point towards smaller, transient filopodia, which do not exhibit ARPC1B-mVenus localization. (**C**) Confocal

projections of embryos expressing *Tg(fli1a:GAL4FF)*; *Tg(UAS:LIFEACT-EGFP)*; *Tg(kdrl:NLS-mCherry)* treated with 100 µM CK666 (Arp2/3 inhibitor) or DMSO from 23 to 29 hpf. Arrows point towards short/truncated ISVs. (**D**) Quantification of truncated ISVs in CK666 treated embryos. N = 11 embryos (DMSO), N = 10 embryos (CK666). P-value calculated by Mann-Whitney test. Scale Bars: 30 µm (C), 20 µm (A, B), 10 µm magnification in (B).

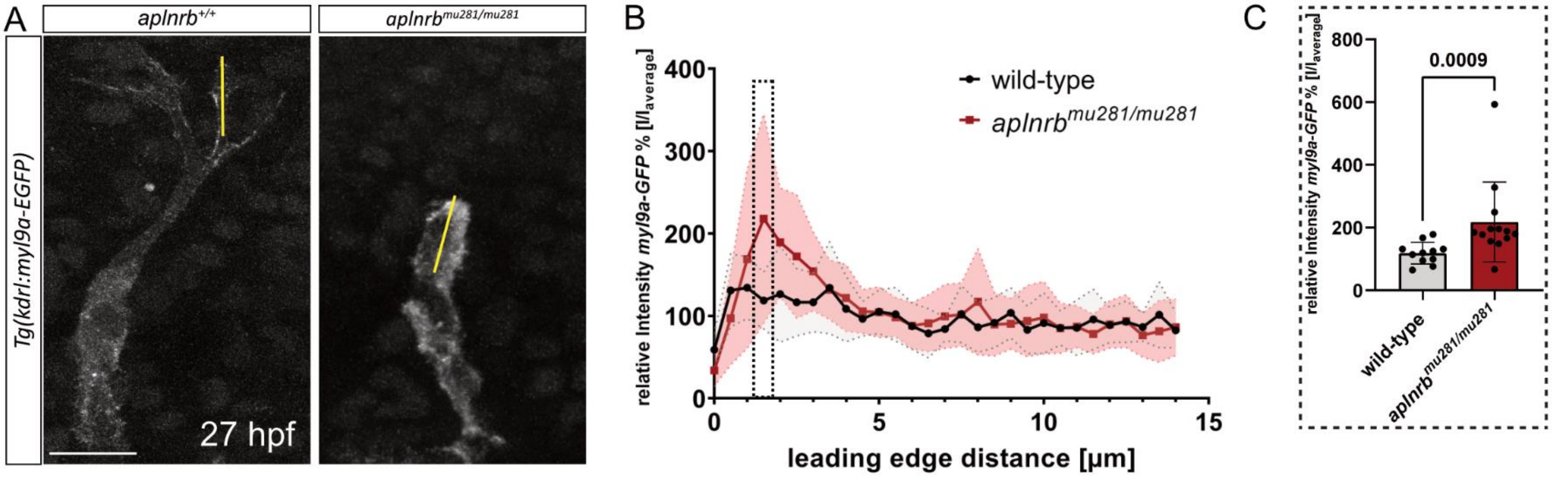

fig. S10: *aplnrb* mutants exhibit mislocalized myosin.
(**A**) Confocal projections of *Tg(kdrl:myl9a-EGFP*) expressing *aplnrb*$^{wt/wt}$ or *aplnrb*$^{mu281/mu281}$ embryos. Lines for line-graph measurements in (A) indicated by the yellow line. (**B**) Line graph depicting normalized fluorescence intensity of myl9a-EGFP. Box with dashed line marks the intensity at 1,5 µm from the leading edge of the cell in (**C**). *aplnrb*$^{wt/wt}$ n = 11 tip cells of N = 4 embryos; *aplnrb*$^{mu281/mu281}$ n = 13 tip cells of N = 6 embryos. P-value calculated by Mann-Whitney test. Scale bar: 20 µm.

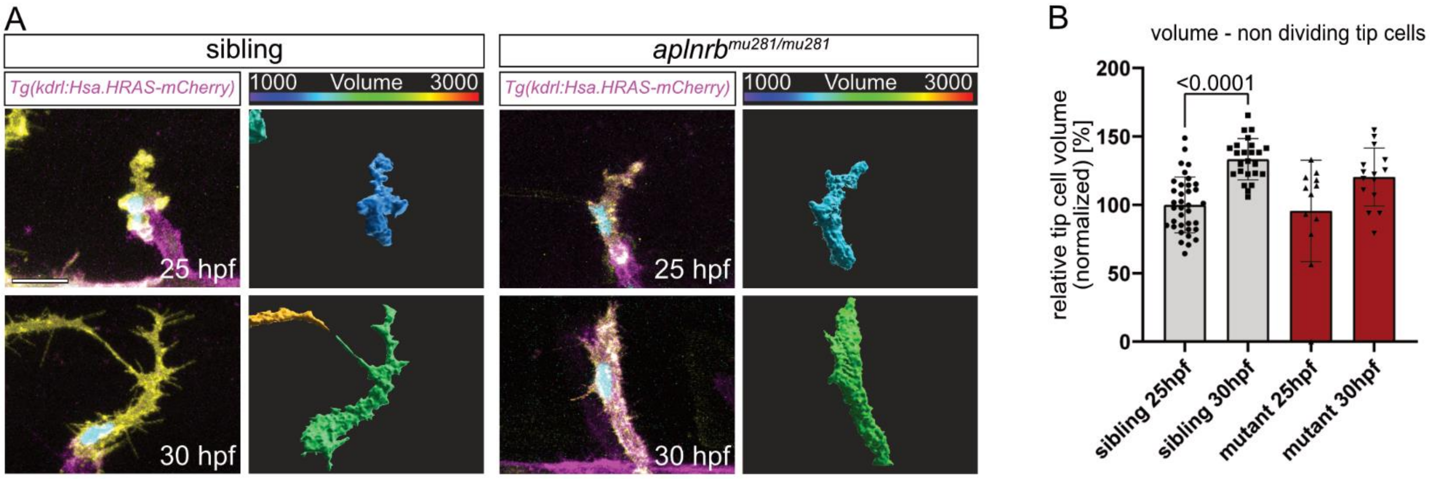

fig. S11: Tip cells expand their volume over time in wild-type and *aplnrb* mutant embryos. (**A**) Confocal images of individually labelled tip cells in siblings and *aplnrb*$^{mu281/mu281}$ embryos at 25 and 30 hpf. Surfaces are color-coded for cell volume. (**B**) Quantification of the tip cell volume. Siblings: 25 hpf n = 36 tip cells, 30 hpf n = 24 tip cells; *aplnrb*$^{mu281/mu281}$ 25 hpf n = 12 tip cells, 30 hpf n = 15 tip cells; P-values calculated by ANOVA with Kruskal-Wallis test. Scale bar: 20 µm.

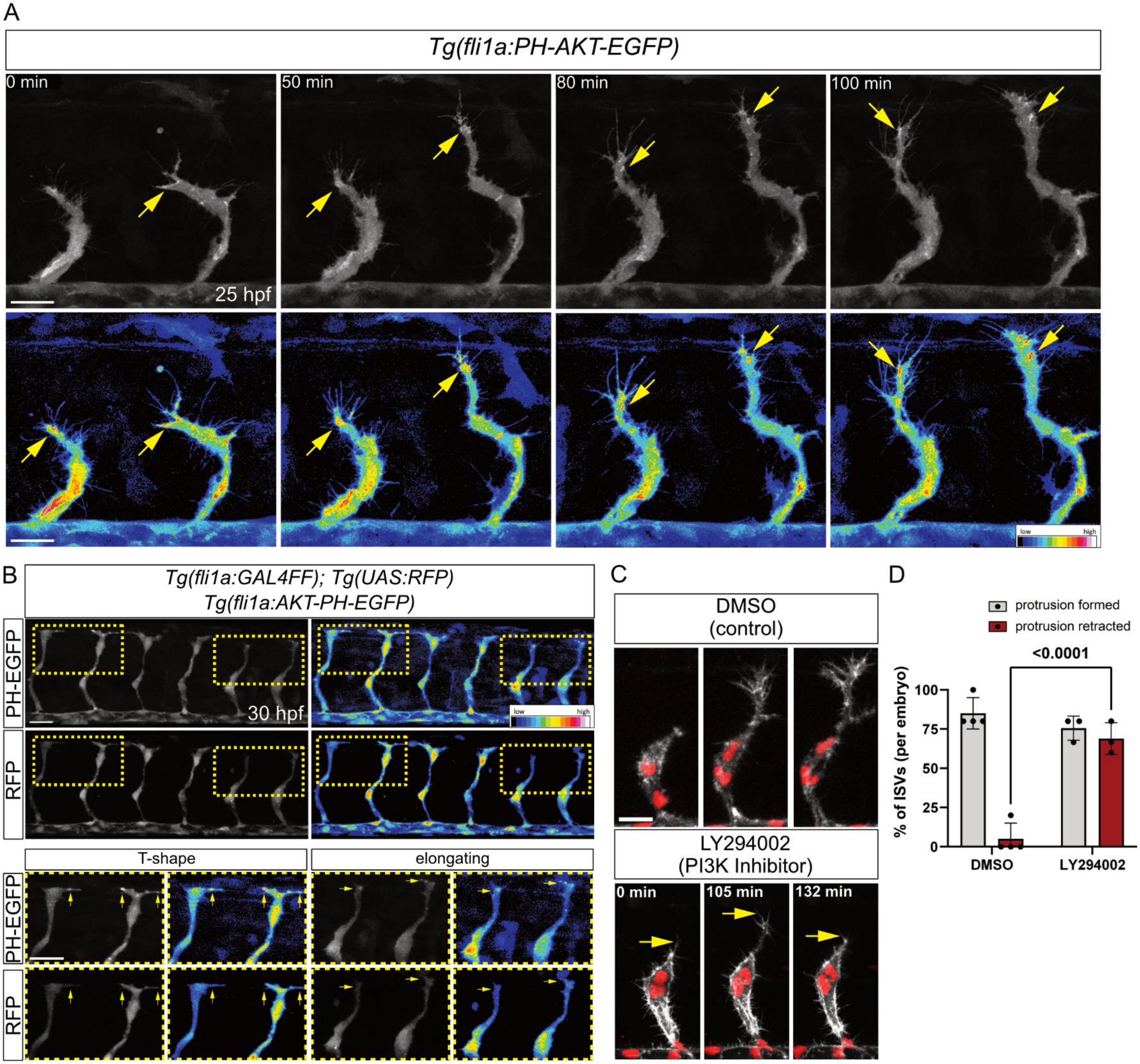

fig. S12: Characterization of the *Tg(fli1a:AKT-PH-EGFP)* transgenic line and protrusion retraction after PI3K inhibition. (**A**) Still images from a confocal time-lapse movie of a *Tg(fli1a:PH-AKT-EGFP)* embryo. Arrows point towards strong signals of PH-AKT-EGFP at the cell front. (**B**) Confocal projections of *Tg(fli1a:GAL4FF)*; *Tg(UAS:RFP)*; *Tg(fli1a:PH-AKT-EGFP)* expressing embryo depicting localization of AKT-PH-EGFP in T-shape and elongating

tip cells in comparison to RFP. (**C**) Still images from time-lapse movies of *Tg(fli1a:GAL4FF)*; *Tg(UAS:LIFEACT-EGFP)*; *Tg(kdrl:NLS-mCherry)* expressing embryos. Embryos were treated with 25 µM of the PI3K inhibitor LY294002 or DMSO (control) from 23 hpf to 30 hpf. Arrows point towards the forming and retracting protrusion after the LY294002 treatment. (**D**) Quantification of protrusions that formed and retracted in tip cells after LY294002 or DMSO treatment. Corresponding to (C). DMSO N = 4 embryos, LY294002 N = 3 embryos; 4 ISVs per embryo observed. P-values calculated by ANOVA with Bonferroni correction. Scale Bars: 20 µm (A), (C), 30 µm (B).

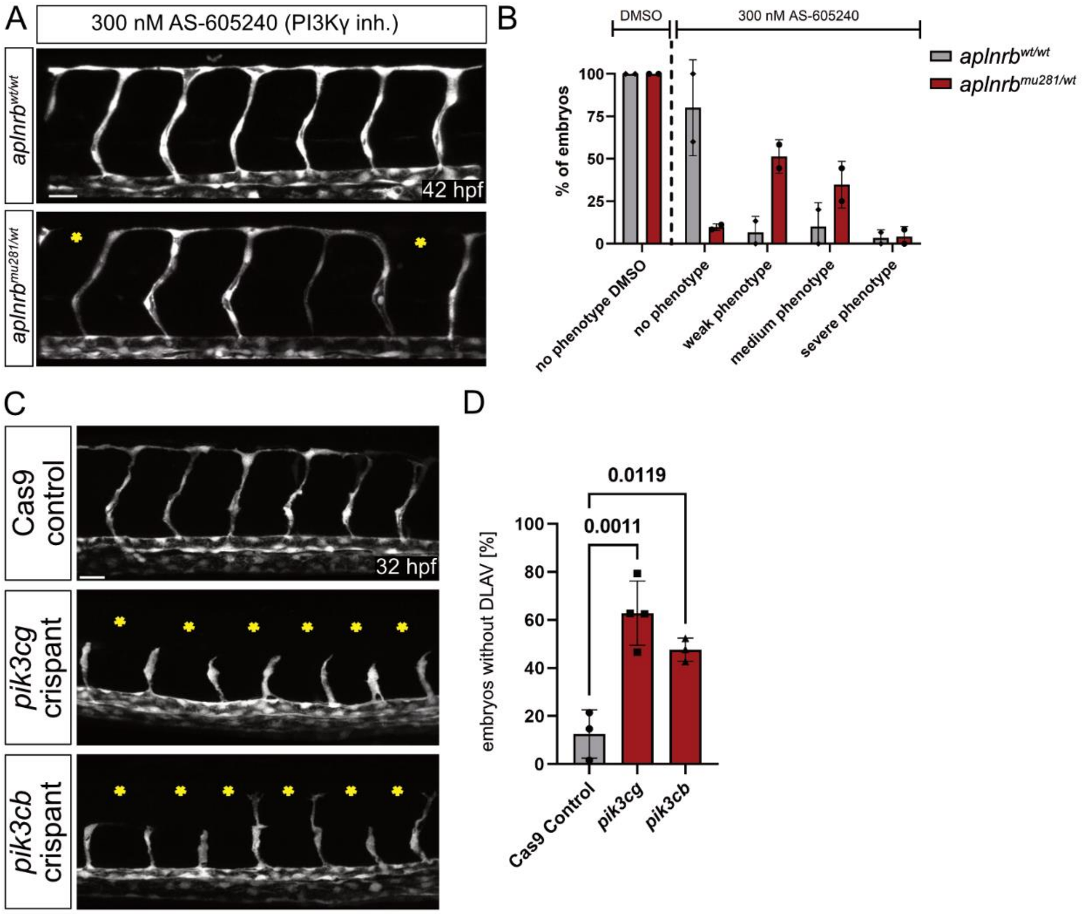

fig. S13: PI3K beta and gamma are required for ISV formation. (**A**) Confocal projections of *Tg(fli1a:EGFP)* expressing *aplnrb*$^{wt/wt}$ or *aplnrb*$^{wt/mu281}$ embryos treated with 300 nM AS-60524 or DMSO from 20 to 42 hpf. Asterisks point towards missing DLAV segments. (**B**) Analysis resulted in different phenotypic categories according to the extent of ISV development. AS-60524: *aplnrb*$^{wt/wt}$ N = 18 embryos; *aplnrb*$^{mu281/wt}$ N = 21 embryos. DMSO: *aplnrb*$^{wt/wt}$ N = 17 embryos; *aplnrb*$^{mu281/wt}$ embryos N = 9 embryos. (**C**) sgRNA injected embryos against indicated catalytic PI3K subunits. Asterisks point towards missing DLAV segments. (**D**) Quantification of the percentage of embryos missing DLAV segments. N = 4 experiments; per experiment and condition at least 30 embryos were screened. P-values calculated by ANOVA with Tukey's test. Scale bars 30 µm.

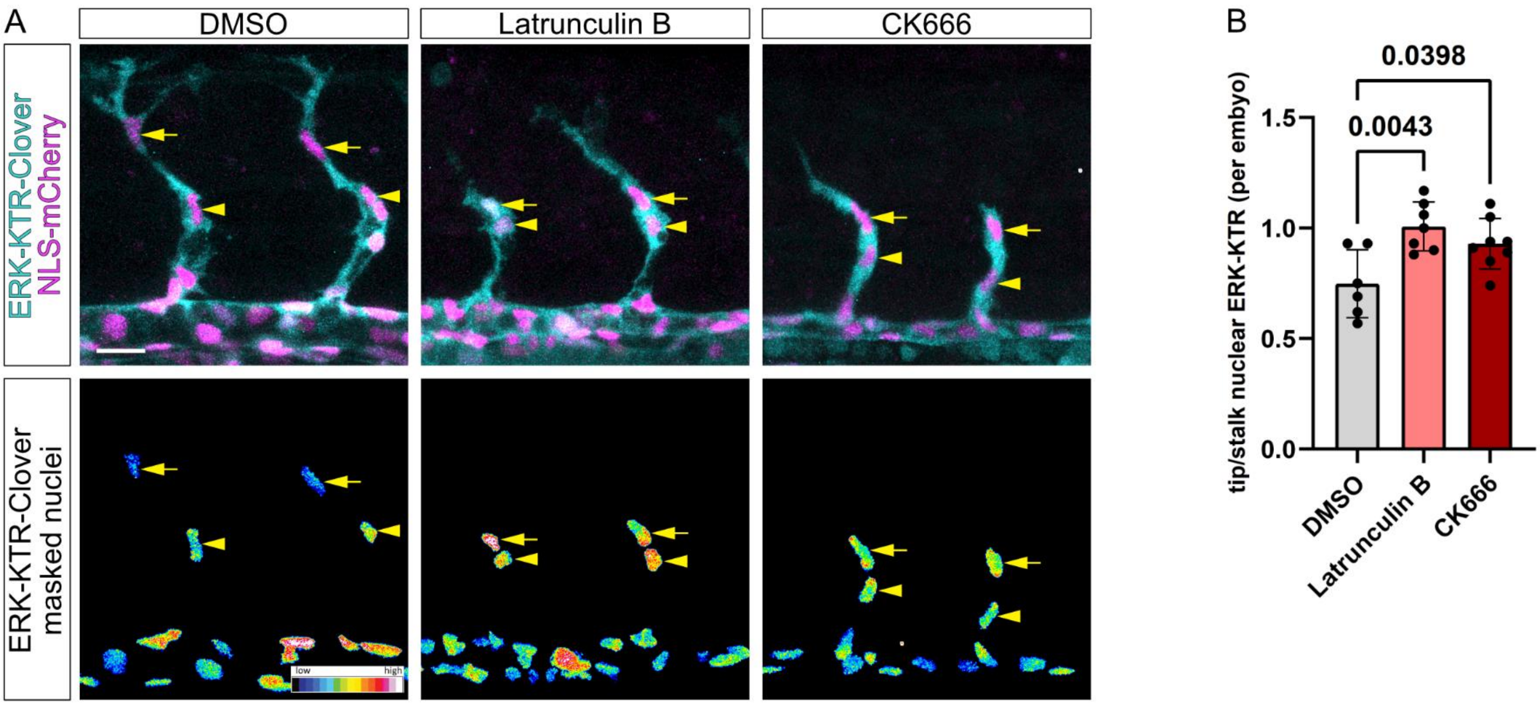

fig. S14: Inhibition of actin polymerization equalizes ERK activity in tip and stalk cells. (**A**) Representative confocal projections of *Tg(fli1aep:ERK-KTR-Clover)*; *Tg(kdrl:NLS-mCherry)* embryos at 27 hpf treated with DMSO (control), 375 nM Latrunculin B or 100 µM CK666 from 24 to 27 hpf. Arrows point to tip cell nuclei; arrowheads point to stalk cell nuclei. Masked nuclei channel color coded for intensity. (**B**) Quantification of nuclear ERK-KTR-Clover intensity ratio of tip / stalk cells. DMSO N = 6 embryos; Latrunculin B N = 7 embryos; CK666 N = 8 embryos. 4 ISVs per embryo quantified. Scale bar 20 µm.

Movie S1.

TgBAC(apln:Venus-PEST) expression during ISV sprouting.

Movie S2.

TgBAC(apln:Cre-ERT2); Tg(-3.5ubb:loxP-EGFP-loxP-mCherry) overview of labeled cells at 5 dpf (360° rotation).

Movie S3.

High-resolution time-lapse of ISV sprouting in *Tg(kdrl:EGFP-CAAX)* of *aplnrb* wild-type vs mutant.

Movie S4.

Filopodia widening and membrane ruffling in ISVs - *Tg(kdrl:EGFP-CAAX) Tg(kdrl:NLS-mCherry).*

Movie S5.

Localization of *Tg(fli1a:ARPC1B-mVenus)* during ISV sprouting and filopodia widening.

Movie S6.

Protrusion retraction in *aplnrb* mutant embryo expressing *Tg(myl9a-EGFP)*.

Movie S7.

Tip cell shuffling during ISV sprouting in *aplnrb* mutants.

Movie S8.

Localization of *Tg(fli1a:PH-AKT-EGFP)* during ISV sprouting.
